# Examining Pubertal and Cognitive Differences in Adolescent Depressive Symptoms Using Drift Diffusion Modeling

**DOI:** 10.1007/s10802-026-01493-4

**Published:** 2026-08-14

**Authors:** Kelly R. Barry, Natasha Chaku, Lindsay Till Hoyt

**Affiliations:** 1 Department of Psychology, University of Houston, Heyne Building #126, Houston, TX 77204 USA; 2 Department of Psychology, Fordham University, 226 Dealy Hall, New York City, NY 10458 USA; 3 Department of Psychological and Brain Sciences, Indiana University, 1101 E. 10th St., Bloomington, IN 47405-7007 USA

**Keywords:** Adolescence, Depression, Emotion regulation, Executive functioning, Drift Diffusion Modeling, Puberty

## Abstract

Risk for depression increases during adolescence, reflecting a constellation of cascading interactions between affective, biological, and cognitive factors. Drift diffusion modeling (DDM) can clarify the cognitive mechanisms underlying risk for depression by decomposing cognitive task performance into latent components, such as drift rate, boundary separation, and non-decision time. However, limited research has applied DDM to adolescent depression or examined whether these cognitive processes interact with pubertal timing, a key biological risk factor. Participants were 103 racially/ethnically and socioeconomically diverse youth (M_age_ = 12.58, 48.5% female) who completed cognitive and affective inhibitory control tasks as well as self-report measures of pubertal development and depressive symptoms. DDM parameters were derived using the EZ diffusion model, and moderation analyses tested whether DDM parameters interacted with pubertal timing to predict depressive symptoms. Earlier pubertal timing was associated with greater depressive symptoms across all models. Boundary separation, but not drift rate or non-decision time, significantly interacted with puberal timing in both the Stroop and Emotional Stroop tasks. Specifically, earlier development was associated with higher depressive symptoms only for youth with high boundary separation, potentially reflecting more cautious or effortful decision-making styles. These findings suggest that both cognitive and biological vulnerabilities both contribute to risk for depression during early adolescence. By identifying some of the specific latent decision-making processes associated with depressive symptoms, DDM may provide insight into cognitive features of depression like rumination, indecision, and increased error monitoring.

**Supplementary Information:**

The online version contains supplementary material available at 10.1007/s10802-026-01493-4.

## Introduction

Depressive symptoms increase significantly during adolescence, with approximately 36% of girls and 13% of boys experiencing at least one depressive episode between ages 12 and 17 (Breslau et al., 2017). Although multiple, cascading mechanisms contribute to the emergence of adolescent depression, few theoretical models integrate affective, biological, and cognitive factors within a developmentally grounded framework. The Affective, Biological, and Cognitive (ABC) model of depression (Hyde et al., 2008) offers one such approach: It describes how depression may arise from multiple vulnerabilities (e.g., early pubertal timing, cognitive vulnerabilities) that co-occur and interact in sex-specific ways (Hyde & Mezulis, 2020). Thus, guided by the ABC model, the present study investigates whether associations between pubertal timing and depressive symptoms are moderated by individual differences in cognitive and affective functioning during adolescence.

## Pubertal Timing and Depression

Puberty is a defining developmental milestone of adolescence, marking the transition to reproductive maturity. Pubertal timing, defined as the age at which youth begin puberty relative to same-aged, same-sex peers, varies widely, with some girls beginning as early as ages 8–9-years-old and some boys as late as 13–14-years-old (Dorn & Beltz, 2023). Variation in pubertal timing is associated with social and emotional risks, particularly for earlier developing youth (Graber, 2013). For girls, earlier pubertal timing has consistently been linked with elevated depressive symptoms during adolescence and adulthood (Hoyt et al., 2020; Hyde & Mezulis, 2020; Ullsperger & Nikolas, 2017). For boys, findings are more mixed: Both earlier and later pubertal timing has been associated with elevated depressive symptoms across studies (see Stumper & Alloy, 2023 for review).

Several mechanisms have been proposed to explain these associations. Sex differences in pubertal hormone expression, primarily estradiol in girls and testosterone in boys, may contribute directly to increased depression risk in girls (Graber, 2013; Schulz et al., 2009). At the same time, the prefrontal (i.e., orbitofrontal and medial prefrontal cortices) and limbic (i.e., the amygdala, ventral striatum) brain regions supporting social, emotional, and cognitive processing are sensitive to the hormonal changes that occur during puberty and continue to develop across adolescence (Ladouceur et al., 2019; Pfiefer & Allen, 2021). Indeed, pubertal timing may influence the pace and coordination of these neurodevelopmental processes, shaping how youth process, evaluate, and respond to cognitive and emotional information (Brieant et al., 2026; Chaku et al., 2024; Ravindranath, 2024). For example, one study found that earlier pubertal timing was associated with better attentional control in girls and boys, but worse self-control in girls only (Chaku & Hoyt, 2019), suggesting that earlier pubertal timing may be associated with sex-specific differences in cognitive and affective processing which, in turn, may influence risk for depression. Alternatively, early maturing youth may lack the emotional and cognitive resources to manage the social demands associated with more adult-like appearances, increasing risk for depressive symptoms through increases in psychosocial stress (Oppenheimer et al., 2018). For example, early maturing youth may have greater difficulty regulating their emotional responses in socially demanding contexts, which can place increased demands on the cognitive processes implicated in depression (Alloy et al., 2016). Together, these perspectives suggest that pubertal development, and its associations with depression, cannot be understood in isolation, but must be considered alongside the cognitive, neurobiological, and psychosocial changes that also characterize adolescence (Pfeifer & Allen, 2021; Hyde, 2020).

## Investigating Cognitive and Affective Influences on Depression

Despite known risks associated with earlier pubertal timing (Dorn & Beltz, 2023), many early developers do not experience depressive symptoms, prompting interest in individual characteristics that may buffer or amplify this risk. Although adolescence is a period of rapid cognitive development (Pfeifer & Allen, 2021), some youth may exhibit cognitive or affective vulnerabilities that interact with puberty to confer risk for depression (Hyde, 2020). A key dimension of interest is inhibitory control, a higher-order cognitive process central to goal-directed behavior that involves the capacity to monitor and modulate responses to task-relevant vs. irrelevant stimuli.

Inhibitory control has demonstrated robust associations with adolescent depression. Inhibitory control plays a central role in shaping adolescents’ ability to disengage from negative information, interrupt repetitive thinking, and flexibly redirect attention towards adaptive activities (Wagner et al., 2015). Deficits in inhibitory control are also linked to specific depressive symptoms, including negative attentional biases (Peckham & Johnson, 2018) and ruminative thought patterns (Stange et al., 2017). Inhibitory control may be relevant not only to cognitive symptoms of depression, but also more broadly across difficulties that characterize depressive symptoms during adolescence. For example, lower inhibitory control has also been associated with difficulties concentrating and increased behavioral dysregulation among adolescents with major depressive disorder (Vijayakumar et al., 2016).

Similarly, poor *affective* inhibitory control, the ability to inhibit responses when faced with emotionally salient stimuli, may contribute to depressive symptoms through interpersonal mechanisms: dysregulated emotional expression can trigger excessive reassurance seeking (Clayton et al., 2021), inappropriate affective responses in social contexts (Maalouf & Brent, 2012), and ultimately peer rejection or conflict (Do et al., 2023). Conversely, stronger affective inhibitory control abilities are thought to promote resilience, enabling adolescents to cope with stressors via reappraisal (Shapero et al., 2019), attentional shifting (Bendezú et al., 2022), and engaged coping strategies (Schäfer et al., 2017).

Critically, research on how pubertal timing and cognition jointly predict depression has yielded mixed and sometimes contradictory findings. For example, some research demonstrates that the effect of earlier pubertal timing on depressive symptoms is only evident among adolescents with maladaptive cognitive styles such as negative attribution bias or negative urgency (Hamilton et al., 2014; Mendle et al., 2020). At the same time, however, other work suggests that pubertal timing is an independent predictor of depressive symptoms regardless of baseline symptoms, cognitive vulnerabilities, and other sociodemographic factors (Copeland et al., 2019; Ullsperger & Nikolas, 2017). One possible explanation for inconsistencies seen across the literature is that prior research has largely focused on self-reported cognitive styles, which may represent downstream consequences of depressive symptoms rather than neurocognitive processes such as inhibitory control which may represent transdiagnostic mechanisms shaping subsequent cognitive vulnerabilities. To better understand these cognitive mechanisms, there is a need to examine individual differences in adolescent cognition using computational models like drift diffusion modeling (DDM), while simultaneously accounting for developmental contexts, such as puberty.

## New Models for Studying Links Between Puberty, Cognition, and Depression

Traditional indices of inhibitory control (e.g., accuracy or reaction time) provide coarse markers of performance and may obscure the distinct cognitive processes that underlie behavior. Indeed, inconsistencies in performance could stem from variations in cognitive task paradigms and analytic approaches. For example, inhibitory control can be assessed using different tasks (e.g., Stroop, Flanker), and even within a single task, performance could be evaluated through multiple metrics such as reaction time, accuracy, trial type (e.g., congruent vs. incongruent), or composite scores (Epp et al., 2012; Khng & Lee, 2014). Thus, worse task performance could reflect different underlying processes such as slower information processing, more cautious responding, or sensitivity towards emotionally salient information, mechanisms that are theoretically distinct but behaviorally similar.

To address these inconsistencies, analytic frameworks such as drift diffusion models (DDM) have been used to elucidate the precise cognitive mechanisms underlying different tasks. DDM is a sequential sampling model that decomposes decision-making tasks into distinct parameters: boundary separation (i.e., amount of information required to make a decision), drift rate (i.e., efficiency of information processing), and non-decision time (i.e., time spent on perception and motor execution; Farrell & Lewandowsky, 2018; Myers et al., 2022). Thus, within the DDM framework, once a stimulus is presented and encoded (non-decision time), evidence is gradually accumulated (drift rate) until a response threshold (or boundary separation) is reached.

DDM enables a better understanding of cognitive processes associated with risk for depression compared to traditional measures of accuracy or reaction time alone which cannot distinguish the precise cognitive mechanisms underlying responses (White et al., 2022; Servant et al., 2015). In other words, two adolescents may show similar levels of accuracy or reaction time but arrive at that performance through different cognitive pathways. For instance, slower reaction times may reflect inefficient evidence accumulation, greater response caution, or slower perceptual/motor processing, each of which has different theoretical implications for understanding risk for depressive symptoms. By decomposing task performance into separable cognitive parameters, DDM thus provides a precise approach for identifying which aspects of cognition are associated with pubertal development and depressive symptoms.

DDM has recently been used to investigate depression-related changes in both cognitive and affective domains. Lower drift rate (or slower evidence accumulation) and wider boundary separation (i.e., more cautious responding) have been associated with increased depressive symptoms in adults (Lawlor et al., 2020; White et al. ,^2010^; Pe et al., 2013), though not all findings are consistent (see Grange, 2022). Although this evidence is promising, few studies have explored links between cognition and risk for depression in adolescent samples. For example, longer nondecision time and slower drift rate (i.e., how fast evidence accumulation occurred) have been associated with higher genetic risk scores for psychopathology in youth (Pedersen et al., 2023) and more internalizing problems (Warren et al., 2025) but links with pubertal development are still unclear.

## The Current Study

Given the complicated etiology of youth depression and increases in symptomology during adolescence, the present study aims to examine biological and cognitive-affective factors in a racial/ethnic and socioeconomically diverse sample of 103 adolescents from a large urban area. Guided by the ABC model of adolescent depression (Hyde et al., 2008) and using novel modeling strategies (Wagenmakers et al., 2007), this study examines (1) how sex, pubertal timing, and cognitive-affective factors were associated with depressive symptoms; and (2) how interactions between pubertal timing and cognitive-affective factors were associated with depressive symptoms. We hypothesized that girls would report higher depressive symptoms than boys and that earlier pubertal timing would be associated with increased depressive symptoms. We also expected that slower drift rates and larger boundary separation would be associated with increased risk for depressive symptoms. Further, we expected that each cognitive-affective parameter would interact with pubertal timing to predict risk for depressive symptoms; however, given limited research in adolescent samples, we did not make hypotheses on the direction of effects.

## Method

### Participants and Procedure

Participants were recruited as part of a larger study exploring the association between physical exercise and cognitive skills in a community sample of urban adolescents. Families were recruited through online social media advertisements, word of mouth referrals, and flyers in the local community. Parents completed an online screening survey to determine eligibility, which included: adolescent participants were between 9 and 16 years old, living in New York City, able to engage in 20 min of moderate aerobic activity, and did not have a documented developmental disability. Eligible families were invited to campus to participate in a 90-minute experimental session. After consent and assent were obtained, caregivers completed survey measures in a private room while youth participated in a series of self-report assessments, anthropometric measures, saliva samples, and computerized executive functioning (EF) tasks in the lab. Additionally, youth also played a video game for 20 min; this data was not used in the present study. Families were paid $50 for their participation. All study procedures were approved by the Institutional Review Board at Fordham University.

### Measures

#### Depressive Symptoms

Youth depressive symptoms were measured via self-report using an abbreviated version of the Center for Epidemiological Studies – Depression (CES-D-8; Radloff, 1977). The CES-D 8-item scale is a reliable and valid measure of depressive symptomatology in child and adolescent populations (Turvey et al., 1999). Example items included “I felt that I could not shake off the blues.”, “I felt lonely.”, and “I had crying spells.”. Youth were instructed to think about the past week when responding to the items on a 4-point scale. Responses ranged from 1 = *Never or very rarely* to 4 = *Most of the time*. In the present sample, the CES-D had an internal consistency of α = 0.75.

#### Pubertal Timing

Pubertal timing was calculated using the youth report of the Pubertal Development Scale (PDS; Petersen et al., 1988), a widely used measure of puberty that is correlated with biological measures of development (Shirtcliff et al., 2009). The PDS consists of three sex-neutral items measuring growth in height, body hair growth, and skin changes as well as four sex-specific items (girls: breast development, first menses; boys: voice changes, facial hair growth). Responses for each item ranged from 1 = *Growth has not yet begun* to 4 = *Growth seems complete*. For girls, menarche was recoded as 1 = *No* and 4 = *Yes*, and if endorsed, participants also reported their age at first menses. Items were averaged to create a continuous composite score.

Following previous work (Mendle et al., 2019), the composite score of pubertal status was then standardized within sex and regressed onto age to create an indicator of pubertal timing. A positive score indicated *earlier* pubertal development (i.e., more mature compared to same-age, same-sex peers) and a negative score indicated *later* pubertal development (i.e., less mature compared to same-age, same-sex peers). Pubertal timing was treated as a continuous variable in all statistical analyses, however, categorical labels such as “earlier” or “later” timing were used for descriptive interpretation and visualization. Internal consistency was acceptable for both sex-specific PDS scales, with omega total values of ω = 0.77 for girls and ω = 0.73 for boys.

#### Cognitive-Affective Tasks

The Stroop Color Word task (Golden, 1978) and the Emotional Stroop (Williams et al., 1996) were created and displayed using Inquisit 5 software (Millisecond Software, 2016). The Stroop Color Word task is a common measure of inhibitory control. In this computerized task, participants were presented with 100 trials of a color word (red, yellow, blue, green), printed in a colored font (red, yellow, blue, green). Participants pressed the corresponding letter to indicate the *color* of the written word, not the word itself. About half of the trials were congruent (e.g., the word and the color font matched: “red” written in red colored font), and half were incongruent (e.g., the word and the color font did not match, “red” written in yellow colored font). Reaction time (in milliseconds) and accuracy were recorded for each trial using Inquisit 5 software.

The Emotional Stroop is a measure commonly used to examine cognitive affective-related inhibitory processes. In this computerized task, participants were presented with 100 trials, each of which contained pictures of faces displaying happy, sad, or angry emotional expressions. Overlaid on each image, was an emotion word (“happy”, “sad”, “angry”). Participants were required to press the corresponding key (*Good* for “happy” and *Bad* for “sad” or “angry”) to indicate the emotion word presented to them, while ignoring the emotion displayed on the face in the picture. Unlike the Stroop Color Word task, about 75% of the Emotional Stroop trials were congruent (e.g., happy facial expression and positive emotion word displayed simultaneously) and only 25% were incongruent (e.g., happy facial expression and negative emotion word displayed simultaneously). Reaction time (in milliseconds) and accuracy were recorded for each trial using Inquisit 5 software.

### Sex

Sex at birth (0 = female, 1 = male) was included given well-established sex differences in the prevalence and developmental course of adolescent depression, as well as differences in pubertal development and cognitive processes that may influence risk for depressive symptoms (Hyde et al., 2008; Hyde & Mezulis, 2020). All participants identified as cisgender, with their gender identify aligning with their sex assigned at birth. Accordingly, we use the terms girls and boys throughout the manuscript when referring to participants.

#### Covariates

Parent depression (CES-D-8; (ω = 0.89); Radloff, 1977), parent education (0 = less than a college degree, 1 = college degree or higher), income-to-needs ratio, and child race/ethnicity were included as covariates in all models. All covariates were selected based theoretical relevance for the development of depressive symptoms in adolescence. Specifically, parent depressive symptoms may represent a significant risk factor for youth depression through both genetic and environmental pathways (Hankin, 2015). Parent education and income-to-needs ratio were included as indicators of socioeconomic context, which has been linked to adolescent risk for depressive symptoms (Xiang et al., 2024). Finally, child race/ethnicity was included given systematic inequities in social experiences that racially diverse adolescents face which could contribute to risk for depressive symptoms (Wilson & Dumornay, 2022). All covariates were assessed via parent report.

### Analytic Plan

All analyses were conducted in R (4.0.0, R Core Team, 2021) and proceeded in three steps. First, we computed descriptive information to describe any initial sex differences using the psych package in R (Revelle, 2025). Then, trial-level data from the Stroop and Emotional Stroop were preprocessed in preparation for modeling the DDM parameters using the tidyverse and dplyr packages (Wickham et al., 2019, 2023). Specifically, we converted reaction times into seconds (s) and then excluded implausibly fast responses (< 300 ms) and excessively slow responses (> 15 s) following convention (Ratcliff, 1993). Additionally, we calculated participant-specific upper bounds (median + 3 × interquartile range) and excluded trials exceeding this threshold to reduce the influence of outliers (Tukey, 1977). Collectively, these preprocessing steps resulted in minimal data loss (< 3% of trials in total). Following preprocessing, for each participant and task, we calculated accuracy as the proportion of correct responses across all valid trials, and mean reaction time and variance using correct trials only.

Then, we used summary statistics from the cognitive tasks to estimate a DDM model using the EZ diffusion model (Wagenmakers et al., 2007). The EZ diffusion model is a simplified version of the DDM model that does not require full hierarchical diffusion model estimation and is well-suited for cognitive tasks that have fewer trials (e.g., < 100) and lower error rates (Lerche et al., 2017). The EZ diffusion model estimates parameters using mean reaction times, variance of reaction time, and accuracy rather than trial-level data, and assumes a binary decision process (correct vs. incorrect responses) and no starting point bias (z fixed at 0.5). In the EZ diffusion model, reaction time and accuracy from congruent and incongruent trials were modeled simultaneously. Thus, the parameters reflect the overall evidence accumulation process underlying task performance rather than condition or task-specific effects, consistent with prior work (Tomlinson et al., 2025).

The EZ model was fit using openly available R scripts and the rtdists package (v0.11.5; Singmann et al., 2018; Weigard & Sripada, 2021). It derives three (rather than four) parameters for each task: drift rate (*v*), boundary separation (*a*), and nondecision time (*Ter*) because the starting point (z) is fixed. Drift rate is an estimate of information uptake; a higher value indicates more efficient information processing. Boundary separation is an estimate of how much information an individual requires to make a decision; higher values indicate more conservative (or less impulsive) decision making. Nondecision time is an estimate of all nondecision-related processes (e.g., stimuli encoding, motor preparation); higher values may indicate more complex or difficult motor responses.

Finally, we conducted hierarchical regression analyses to examine associations between DDM parameters, pubertal timing, sex, and risk for depressive symptoms using the *lm* package to conduct the analyses and sjPlot package to visualize the results (Lüdecke, 2024, version 2.8.17). Each DDM parameter (drift rate [*v*], boundary separation [*a*], and non-decision time [*ter*]) from each cognitive task (Stroop Color Word, Emotional Stroop) was examined in a separate model. In the first set of models, we examined the main effects of sex, pubertal timing, and each DDM parameter, resulting in 6 models. In the second set of models, we investigated interactions between pubertal timing and each DDM parameter, resulting in 12 total models. Finally, as an exploratory analyses, we investigated three-way interactions between pubertal timing, each DDM parameter, and sex. Each model controlled for age, income to needs, parent’s education, race/ethnicity, parent depression. Standard OLS regression diagnostics indicated that model assumptions were adequately met, with no major violations of linearity or homoscedasticity based on residual plots, approximately normally distributed residuals, and low multicollinearity across predictors (i.e., variance inflation factors below 2.5).

Given the large number of models, type I error inflation was adjusted for using the Benjamini-Hochberg (BH) False Discovery Rate (FDR) procedure against a nominal alpha of 0.05 and a false discovery rate of 0.25 (Benjamini & Hochberg, 1995). Corrected *p* values are reported in text. Significant interactions (*p* < .05*)* that survived FDR corrections were probed using the Johnson-Neyman technique. This was used to determine the region of significance (that is, the range of moderator values at which the effect of pubertal timing on depression was significant). Interactions were also plotted at one standard deviation above, at, and below the mean to aid interpretation.

## Results

Participant characteristics and bivariate correlations for key variables are presented in Table 1. The final sample consisted of 103 participants (M_age_=12.58; SD = 2.13) 14.56% White, 45.63% Black, 35.92% Hispanic/Latino/Spanish, 3.88% American Indian/Alaskan Native, 11.65% Asian.^1^ As expected, girls reported more depressive symptomatology than boys (*d* = − 0.44) as well as earlier pubertal timing (*d* = − 0.23). On the cognitive tasks, girls tended to have faster drift rates (*d*s: 0.01 - − 0.32) and smaller boundary separations (*d*s: 0.01 − 0.32), indicating more efficient information processing and speedier responding respectively.

**Table 1:** Descriptive statistics and correlations of study variables, separated by sex

1	1. Depressive Symptoms		2. Pubertal timing		3. Stroop drift rate	4. Stroop boundary separation	5. Stroop non-decision time	6. Emotional Stroop drift rate	7. Emotional Stroop boundary separation	8. Emotional Stroop non-decision time	9. Age	10. Income to needs		11. Mother’s education
	-													
2	0.13		-											
3	− 0.03		0.25*		-									
4	0.04		− 0.24*		− 0.46***	-								
5	0.16		− 0.13		− 0.29**	0.23*	-							
6	− 0.10		0.42***		0.48***	− 0.44***	− 0.28**	-						
7	0.16		0.19		0.18	− 0.25**	− 0.19	0.16	-					
8	− 0.22*		− 0.29**		− 0.36***	0.39***	0.22*	− 0.36***	− 0.60***	-				
9	− 0.03		0.70***		0.26**	− 0.30**	− 0.20*	0.51***	0.10	− 0.43***	-			
10	− 0.09		− 0.01		0.20*	− 0.21*	0.10	0.21*	− 0.25*	− 0.08	0.11	-		
11	− 0.05		0.10		0.31**	-21*	− 0.003	0.20	− 0.16	− 0.07	0.12	0.64***		-
*Girls* (*n* = 50)														
*M*	0.47		2.49		0.91	2.42	0.57	1.86	1.59	0.83	12.22	1.73		4.26
*SD*	0.47		0.76		0.47	0.71	0.34	0.71	0.25	017	2.16	1.25		1.95
*Boys* (*n* = 53)														
*M*	0.29		2.34		1.08	2.19	0.57	1.87	1.54	0.75	12.91	2.17		4.60
*SD*	0.37		0.55		0.58	0.76	0.38	0.97	0.27	0.20	2.06	1.48		2.28
*Effect Size (Cohen’s d) for Sex Differences*														
	− 0.44	− 0.23		0.32		− 0.31	0.01	0.01	− 0.17	− 0.43	0.33	0.32	0.15	

Note. Correlations were conducted separately for pairwise relations. 95% confidence intervals are presented in brackets. Following convention, effect sizes for sex differences are positive if men have higher means than women, and negative if women have higher means than men. *M*: Mean; *SD*: Standard deviation

* *p* < .05, ** *p* < .01, *** *p* < .001

**Fig. 1:**
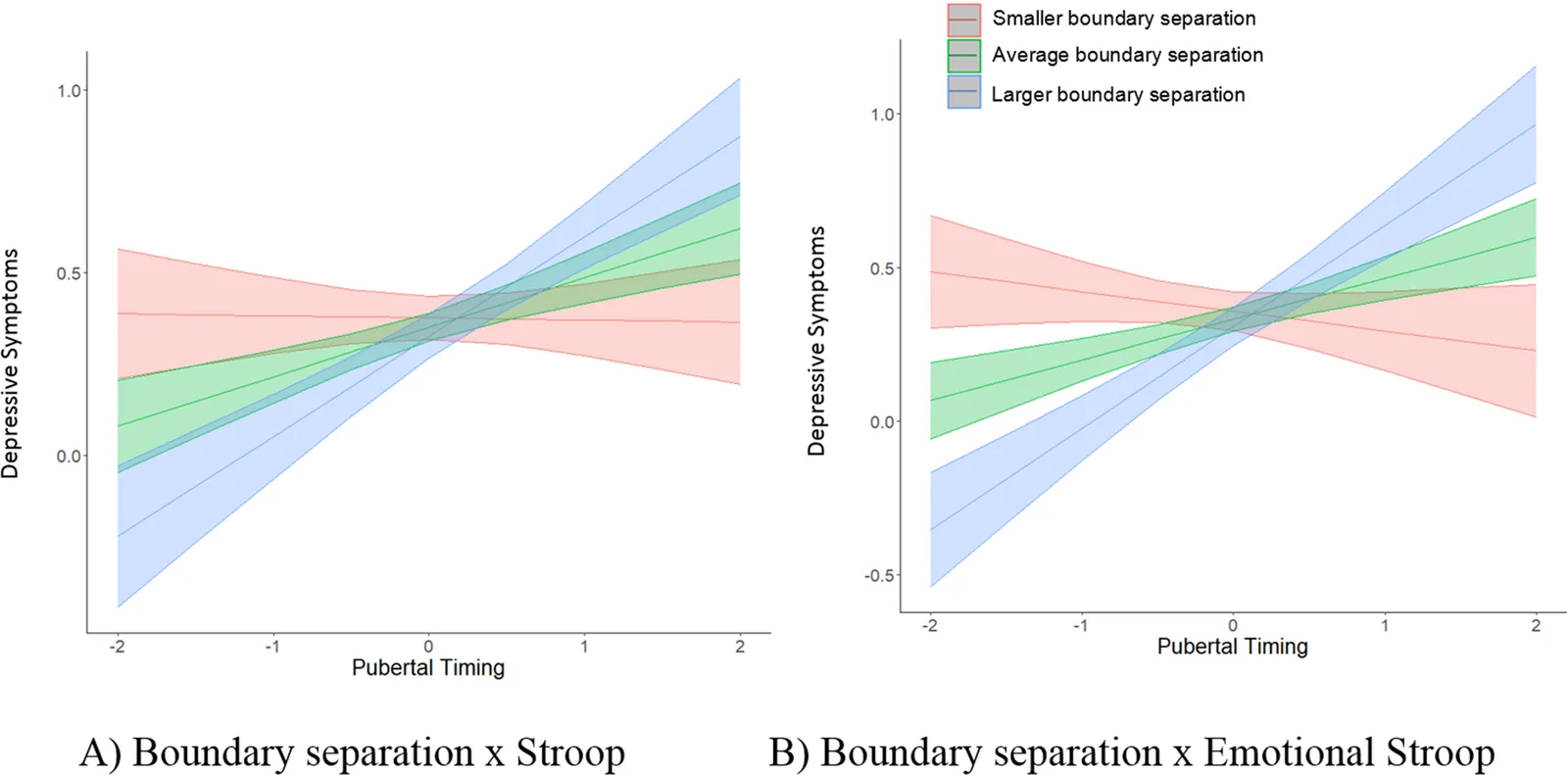
Boundary separation derived from the Stroop Color Word Task (**A**) and Emotional Stroop (**B**) moderated the association between pubertal timing and depressive symptoms. Analysis of conditional effects revealed that early developing youth with larger boundary separation reported more depressive symptoms whereas later developing youth with larger boundary separation reported fewer depressive symptoms. A). The Johnson-Neyman interval for the Stroop revealed that when boundary separation is outside the interval [-3.01, 1.00], the slope of pubertal timing is *p* < .05. The range of observed values of the boundary separation is [-0.28, 2.74]. B). The Johnson-Neyman interval for the Emotional Stroop revealed that when boundary separation is outside the interval [-0.67, 1.83], the slope of pubertal timing is *p* < .05. The range of observed values of the boundary separation is [-0.67, 3.56]. (A) Boundary separation x Stroop (B) Boundary separation x Emotional Stroop

Table 2 presents the main and interaction effects of sex, pubertal timing, and boundary separation for the Stroop and Emotional Stroop. Earlier pubertal timing was associated with more depressive symptoms in both models (Stroop: *B* = 0.13, corrected *p* = .02; Emotional Stroop: *B* = 0.14, corrected *p* = .01), and boys reported lower depressive symptoms than girls though this effect did not survive correction for the Emotional Stroop (Stroop: *B* = − 0.17, corrected *p* = .05; Emotional Stroop: Stroop: *B* = − 0.17, corrected *p* > .05). Although boundary separation was not significantly associated with depressive symptoms, the interaction between pubertal timing and boundary separation was significant in both models (Stroop: *B* = 0.26, corrected *p* = .01; Emotional Stroop: *B* = 0.23, corrected *p* = .003). Figure 1 shows the pattern of results for this significant interaction. Specifically, at larger boundary separations (i.e., when *M*_*drift rate*_ > 1.00), earlier pubertal timing was associated with more depressive symptoms.

**Table 2:** Moderation analyses of pubertal timing x boundary separation on depressive symptoms

	Stroop				Emotional stroop			
	Main effects		Moderation		Main effects		Moderation	
*Coefficient*	*Estimates*	*CI*	*Estimates*	*CI*	*Estimates*	*CI*	*Estimates*	*CI*
Intercept	0.46	-0.08–1.00	0.50	-0.02–1.03	0.21	-0.35–0.77	0.19	-0.35–0.72
Boys	**-0.17** ^*^	**-0.34 – -0.01**	-0.13	-0.30–0.03	-0.15	-0.32–0.02	-0.13	-0.29–0.03
Age	-0.01	-0.05–0.03	-0.01	-0.05–0.03	0.02	-0.03–0.07	0.01	-0.03–0.06
Income to needs	0.04	-0.03–0.11	0.03	-0.04–0.11	0.04	-0.04–0.11	0.03	-0.04–0.10
College graduate	-0.19	-0.39–0.01	-0.16	-0.35–0.04	**-0.21** ^*^	**-0.40 – -0.01**	**-0.21** ^*^	**-0.39 – -0.03**
Black race/ethnicity	0.11	-0.11–0.33	0.10	-0.12–0.32	0.13	-0.09–0.36	0.16	-0.06–0.37
Hispanic, Latino or Spanish race/ethnicity	0.02	-0.19–0.24	0.01	-0.20–0.22	-0.01	-0.22–0.21	-0.02	-0.23–0.19
American Indian or Alaskan Native	0.45	-0.06–0.96	**0.52** ^*^	**0.02–1.02**	0.47	-0.03–0.97	**0.52** ^*^	**0.04–1.00**
Asian race/ethnicity	0.30	-0.02–0.61	0.26	-0.05–0.57	**0.32** ^*^	**0.01–0.62**	**0.35** ^*^	**0.06–0.65**
Pubertal timing	**0.13** ^*^	**0.01–0.25**	-0.13	-0.37–0.12	**0.14** ^*^	**0.02–0.27**	-0.30	-0.64–0.04
Boundary separation	0.01	-0.15–0.18	-0.05	-0.21–0.12	-0.07	-0.19–0.04	-0.03	-0.14–0.08
Pubertal timing x Boundary separation			**0.26** ^*^	**0.05–0.47**			**0.23** ^**^	**0.07–0.40**
Observations	99		99		95		95	
R^2^ / R^2^ adjusted	0.176 / 0.082		0.228 / 0.130		0.212 / 0.119		0.281 / 0.185	

Note: Mother education was collapsed into 0 = Less than college degree, 1 = college degree or higher; White was used as reference group for race/ethnicity; Pubertal timing, positive values indicate earlier pubertal timing, negative values indicate later pubertal timing.

* *p* < .05   ** *p* < .01   *** *p* < .001

The main and interaction effects of sex, pubertal timing and drift rate and of pubertal timing and non-decision time for the Stroop and Emotional Stroop are available in Supplemental Tables S1 and S2 respectively. As in previous models, girls reported increased depressive symptoms in the Stroop models (*Bs* = − 0.18, corrected *p*s = 0.03 − 0.04), but, in the Emotional Stroop, girls reported increased depressive symptoms in the non-decision time model only (*B* = − 0.19, corrected *p* = .03). Similarly, earlier pubertal timing was associated with more depressive symptoms across all models. Drift rate was not significantly associated with depressive symptoms and although shorter non-decision time was associated with more depression, this was only true for the Emotional Stroop (*B* = − 0.67, corrected *p* = .001); no interaction effects were significant.

Two sets of exploratory analyses were conducted. First, all moderation analyses were re-estimated using Bayesian linear regression in R using non-informative priors (package: bayestestR, version 0.17.0; Makowski et al., 2019) given some evidence that Bayesian methods may provide less biased estimates compared to frequentist methods when sample sizes are small (Smid et al., 2020). Accordingly, these Bayesian regression models were used as complementary analyses, to evaluate robustness of the observed effects. Results are provided in supplemental materials; they are largely consistent with the main and interaction effects reported in the main text.

Second, given well known differences in the onset and progression of puberty (Dorn & Beltz 2023) and in depressive symptoms (Hyde, 2020), an exploratory, three-way interaction between puberty, cognition, and sex was estimated for each cognitive-affective parameter. These results were largely consistent with the two-way interactions: The three-way interaction for boundary separation was significant for the Stroop (*B* = − 0.25, corrected *p* = .02) and trending towards significance for the Emotional Stroop (*B* = − 0.11, corrected *p* = .07); plotting the interaction suggested that the effect of boundary separation on the relationship between pubertal timing and depression was primarily driven by girls. There were no significant three-way interactions for drift rate or non-decision time. See the supplemental materials for associated tables and figures.

## Discussion

Grounded in the ABC model of depression (Hyde et al., 2008), this study examined the joint effects of pubertal timing and cognition on depressive symptoms in a racially/ethnically and socioeconomically diverse adolescent sample. Consistent with expectations, sex was associated with depressive symptoms in our sample, with girls reporting more depressive symptoms than boys. Earlier pubertal timing was a robust predictor of depressive symptoms across tasks for both boys and girls, supporting prior research showing increased depressive risk among early developers (Dorn & Beltz, 2023; Mendle et al., 2019; Deardorff et al., 2019). This is consistent with developmental psychopathology models, suggesting that the pubertal transition may be a salient period for the emergence of sex differences in depression.

Our findings also indicated that early-maturing youth with more cautious response styles, across both tasks, may face compounding vulnerabilities. Specifically, cognitive-affective strategies such as increased boundary separation may reflect rumination, slowed processing, or motivational deficits (Pitliya et al., 2022), which could amplify the emotional and social challenges associated with earlier pubertal timing. Exploratory analysis of the 3-way interaction between sex, pubertal timing, and DDM parameters suggested that there were sex differences, with effects strongest among girls. While the current study was underpowered to formally test sex-specific pathways, this is consistent with the ABC model’s emphasis on heightened affective reactivity among girls during adolescence (Hyde & Mezulis, 2020). Future work with larger samples is needed to clarify whether cognitive-affective vulnerabilities operate differently across sexes.

Contrary to expectations, DDM parameters were not independently associated with depressive symptoms, apart from non-decision time for the Emotional Stroop, suggesting that these cognitive-affective processes may not function as uniform or context-independent predictors of depressive symptoms during early adolescence. While previous studies in adults have found associations between slower drift rates (e.g., less efficient evidence accumulation) or greater boundary separation (Castagna & Crowley, 2021; Lawlor et al., 2020; White et al., 2010) and depression, others have failed to replicate these findings (Grange, 2022). The association between non-decision time and depressive symptoms observed here may reflect adolescent-specific processes, such as delayed motor or perceptual engagement particular to emotionally salient contexts. Alternatively, some evidence also suggests that inhibition is better indexed by non-decision time in younger populations (Schuch & Konrad, 2017; Mueller et al., 2021), further highlighting developmental differences in DDM interpretation. This pattern highlights the importance of considering-affective processes as conditional risk factors, rather than being uniformly associated with depressive symptom risk.

The most compelling finding was the interaction between boundary separation and pubertal timing. Because DDM parameters were estimated using information across both congruent and incongruent trials of the Stroop tasks, boundary separation can be interpreted here as a general response style, rather than specific to different trial conditions (i.e., congruent vs. congruent). Greater boundary separation may reflect a more cautious style of responding, which may be particularly relevant when considered alongside pubertal development. Adolescents with both early maturation and more conservative (or cautious) response tendencies, as assessed by greater boundary separation, showed significantly higher depressive symptoms. This finding aligns with prior research (Castagna & Crowley, 2021; Pe et al., 2013) and underscores the importance of contextualizing cognitive-affective traits within developmental processes. From a cognitive perspective, more boundary separation may reflect a tendency toward increased deliberation, heightened sensitivity to conflict or error, or difficulty committing to responses under conditions of ambiguity. In early adolescence, these tendencies may manifest as indecision, over-evaluation of competing information, or reduced cognitive flexibility. When considered alongside early pubertal maturation, which is associated with heightened affective reactivity and ongoing development of prefrontal regulatory systems, these cognitive tendencies may become particularly salient. Early maturing youth may experience a mismatch between increased socioemotional demands and still-developing cognitive-affective control systems, such that more cautious response styles become maladaptive.

Together, these results suggest that both pubertal timing and cognitive-affective response patterns may both be important for identifying adolescents at greatest risk. Early maturing youth who also display heightened caution in decision-making may represent a subgroup characterized by overlapping cognitive-affective characteristics implicated in depression, such as increased deliberation, difficulty disengaging from task-irrelevant information, slowed processing, or decreased motivation (Rifkin et al., 2021; Pitliya, 2022; Meinzer et al., 2014), all of which could compound the socio-affective challenges of puberty. Identifying such profiles may help inform future research that examines whether cognitive-affective processes, such as speed-accuracy balance and evidence accumulation represent modifiable risk factors, which may in turn clarify potential pathways linking early maturation to depressive symptomology.

### Strengths, Limitations, and Future Directions

This study has several strengths, including its use of a racially and socioeconomically diverse community sample and the novel application of DDM to adolescent depressive symptoms. Importantly, the present study contributes to basic understanding of computationally derived cognitive-affective processes associated with depressive symptoms. DDM provides a powerful tool to uncover latent cognitive processes that may underlie risk and resilience across developmental transitions, moving beyond traditional behavioral metrics, such as reaction time and accuracy, providing more mechanistic insight into cognitive-affective functioning during adolescence. Further, we found consistency across our frequentist and Bayesian analyses, which provides additional evidence for the observed interaction between pubertal timing and boundary separation.

However, several limitations merit consideration. First, the cross-sectional design precludes inferences about directionality or developmental change. Longitudinal studies are needed to examine reciprocal influences between cognitive-affective parameters and depressive symptoms across adolescence (Hankin, 2015). Second, despite multiple comparison corrections and Bayesian replication, the sample size had limited statistical power, particularly for higher-order interactions. Future studies with larger samples could better detect small effects and explore sex, as well as gender, differences and potential moderating effects of race/ethnicity, given established differences in pubertal timing for racial/ethnically diverse youth (Swilley-Martinez et al., 2023).

Third, our sample included youth at varying stages of pubertal development. For some participants, the effects of puberty on cognition and emotion may not yet be fully evident. Longitudinal tracking of pubertal and cognitive-affective development will be essential for pinpointing when and how risks emerge (Chaku et al., 2024; Stumper et al., 2020). In particular, future work should examine whether the interaction between early pubertal timing and cautious decision-making is predictive of specific symptom dimensions, such as rumination or anhedonia, and whether these associations differ across developmental windows (McGuire et al., 2019; Krause et al., 2018). In addition to a self-reported measure of pubertal development, future work would benefit from physiological measures, such as Tanner Staging or hormone measurement (Herting et al., 2021; Shirtcliff et al.,, 2009).

Lastly, the field would benefit from greater consistency in how DDM parameters are interpreted and applied across tasks and age groups. In the current study, Stroop Color Word and Emotional Stroop tasks differed in the proportion of congruent and incongruent trials (approximately 50/50 vs. 75/25); accordingly underlying evidence distributions could differ across tasks. For this reason, DDM parameters were estimated within each task and were not directly compared across the tasks. Establishing standardized developmental benchmarks for DDM parameters would improve the translational value of this approach which may facilitate future integration of cognitive-affective modeling into clinical research and assessment (Mendle et al., 2020; Myers et al., 2022). The EZ diffusion model approach provides estimates of drift rate, boundary separation, and non-decision time, but does not model between-trial variability in these processes. Although this simplified approach is appropriate for samples with fewer trials or lower error rates, future work using full hierarchical diffusion models could further characterize variability in cognitive-affective processing across trials. Further, the differences in congruent-incongruent trials between the Stroop tasks used in this study limit direct comparison of the DDM parameters between tasks.

## Conclusion

The present findings suggest that boundary separation, representing cautious response tendencies, moderates links between earlier pubertal timing and adolescent depressive symptoms. These results underscore the importance of integrating developmental context and individual differences in cognitive processing when evaluating pathways to depression. DDM offers a promising lens through which to study cognitive-affective vulnerabilities in youth and may provide a foundation for future studies to examine how these processes intersect with sex and gender, pubertal development, and the emergence of depressive symptoms. This study highlights the value of simultaneously considering pubertal development and cognitive-affective styles in risk assessment. Interventions that both support early maturing adolescents in navigating heightened social demands and address cautious or over-controlled cognitive-affective patterns may reduce vulnerability to depressive symptoms. By using advanced tools, such as DDM, to capture subtle cognitive-affective vulnerabilities, researchers may be better positioned to examine how cognitive processes interact with developmental factors in shaping risk trajectories to developmental stage, ultimately promoting more resilient trajectories for at-risk youth. Future longitudinal and experimental research will be necessary to determine whether these cognitive-affective processes represent mechanisms of risk or markers of vulnerability.

## Electronic Supplementary Material

Below is the link to the electronic supplementary material.

Supplementary Material 1 (DOCX 63.3 KB)

## Data Availability

Datasets and analytic code of the present study are available from the corresponding author on reasonable request.

## Footnotes

1 Race/ethnicity values exceed 100%, as 13.6% individuals in our sample identified as bi- or multi-racial.

## Declarations

### Clinical Trial Number

N/A.

## References

[CR1] Alloy, L. B., Hamilton, J. L., Hamlat, E. J., & Abramson, L. Y. (2016). Pubertal development, emotion regulatory styles, and the emergence of sex differences in internalizing disorders and symptoms in adolescence. *Clinical Psychological Science*, *4*(5), 867–881. 27747141 10.1177/2167702616643008 PMC5061504

[CR3] Bendezú, J. J., Handley, E. D., Manly, J. T., Toth, S. L., & Cicchetti, D. (2022). Psychobiological foundations of coping and emotion regulation: Links to maltreatment and depression in a racially diverse, economically disadvantaged sample of adolescent girls. *Psychoneuroendocrinology*, *143*, 105826. 35700563 10.1016/j.psyneuen.2022.105826 PMC9357119

[CR4] Benjamini, Y., & Hochberg, Y. (1995). Controlling the false discovery rate: a practical and powerful approach to multiple testing. *Journal of the Royal statistical society: series B (Methodological)*, *57*(1), 289–300.

[CR6] Breslau, J., Gilman, S. E., Stein, B. D., Ruder, T., Gmelin, T., & Miller, E. (2017). Sex differences in recent first-onset depression in an epidemiological sample of adolescents. *Translational Psychiatry*, *7*(5), e1139. 28556831 10.1038/tp.2017.105 PMC5534940

[CR84] Brieant, A., Chaku, N., Beck, D., MacSweeney, N., Rakesh, D., & Tamnes, C. K. (2026). Timing and tempo of puberty and neurodevelopment following adversity: A registered report. *Developmental Cognitive Neuroscience*, 101738. 10.1016/j.dcn.2026.101738 PMC13196395 42127658

[CR7] Castagna, P. J., & Crowley, M. J. (2021). Relationship between puberty and inhibitory control: Computational modeling of the drift-diffusion process. *Developmental Neuropsychology*, *46*(5), 360–380. 34283678 10.1080/87565641.2021.1952206

[CR10] Chaku, N., & Hoyt, L. T. (2019). Developmental trajectories of executive functioning and puberty in boys and girls. *Journal of Youth and Adolescence*, *48*(7), 1365–1378. 30989473 10.1007/s10964-019-01021-2

[CR11] Chaku, N., Waters, N. E., & Ahmed, S. F. (2024). Links between socioeconomic position and cognitive and behavioral regulation in adolescence: The role of pubertal development. *Journal of Research on Adolescence*, *34*(4), 1232–1246. 38845091 10.1111/jora.12964 PMC11606269

[CR12] Clayton, M. G., Giletta, M., Boettiger, C. A., & Prinstein, M. J. (2021). Determinants of excessive reassurance-seeking: Adolescents’ internalized distress, friendship conflict, and inhibitory control as prospective predictors. *Journal of Clinical Child & Adolescent Psychology*, *50*(1), 88–96. 31050555 10.1080/15374416.2019.1604234 PMC6825879

[CR86] Copeland, W. E., Worthman, C., Shanahan, L., Costello, E. J., & Angold, A. (2019). Early pubertal timing and testosterone associated with higher levels of adolescent depression in girls. *Journal of the American Academy of Child & Adolescent Psychiatry*, *58*(12), 1197–1206. 30768421 10.1016/j.jaac.2019.02.007 PMC6693999

[CR13] Deardorff, J., Hoyt, L. T., Carter, R., & Shirtcliff, E. A. (2019). Next steps in puberty research: Broadening the lens toward understudied populations. *Journal of Research on Adolescence*, *29*(1), 133–154. https://www.ncbi.nlm.nih.gov/pubmed/30869847 30869847 10.1111/jora.12402 PMC6827435

[CR14] Do, Q. B., McKone, K. M. P., Hamilton, J. L., Stone, L. B., Ladouceur, C. D., & Silk, J. S. (2023). The link between adolescent girls’ interpersonal emotion regulation with parents and peers and depressive symptoms: a real-time investigation. *Development and Psychopathology, 37*(1), 1–15. 10.1017/S0954579423001359 37933501

[CR15] Dorn, L. D., & Beltz, A. M. (2023). Puberty: foundations, findings, and the future.

[CR16] Epp, A. M., Dobson, K. S., Dozois, D. J. A., & Frewen, P. A. (2012). A systematic meta-analysis of the Stroop task in depression. *Clinical Psychology Review*, *32*(4), 316–328. 22459792 10.1016/j.cpr.2012.02.005

[CR17] Farrell, S., & Lewandowsky, S. (2018). *Computational modeling of cognition and behavior*. Cambridge University Press.

[CR18] Golden, C. J. (1978). *Stroop color and word test: a manual for clinical and experimental uses*. Stoelting.

[CR19] Graber, J. A. (2013). Pubertal timing and the development of psychopathology in adolescence and beyond. *Hormones and Behavior*, *64*(2), 262–269. 23998670 10.1016/j.yhbeh.2013.04.003

[CR20] Grange, J. A. (2022). Computational modelling of the speed–accuracy tradeoff: No evidence for an association with depression symptomatology. *Journal of psychiatric research*, *147*, 111–125. 35032944 10.1016/j.jpsychires.2021.12.057

[CR21] Hamilton, J. L., Hamlat, E. J., Stange, J. P., Abramson, L. Y., & Alloy, L. B. (2014). Pubertal timing and vulnerabilities to depression in early adolescence: Differential pathways to depressive symptoms by sex. *Journal of Adolescence*, *37*(2), 165–174. 24439622 10.1016/j.adolescence.2013.11.010 PMC3939064

[CR22] Hankin, B. L. (2015). Depression from childhood through adolescence: Risk mechanisms across multiple systems and levels of analysis. *Current opinion in psychology*, *4*, 13–20. 25692174 10.1016/j.copsyc.2015.01.003 PMC4327904

[CR23] Herting, M. M., Uban, K. A., Gonzalez, M. R., Baker, F. C., Kan, E. C., Thompson, W. K., & Sowell, E. R. (2021). Correspondence between perceived pubertal development and hormone levels in 9–10 year-olds from the adolescent brain cognitive development study. *Frontiers in Endocrinology*, *11*, 549928. 33679599 10.3389/fendo.2020.549928 PMC7930488

[CR24] Hoyt, L. T., Niu, L., Pachucki, M. C., & Chaku, N. (2020). Timing of puberty in boys and girls: Implications for population health. *SSM – Population Health*, *10*, 100549. https://www.ncbi.nlm.nih.gov/pubmed/32099893 32099893 10.1016/j.ssmph.2020.100549 PMC7030995

[CR25] Hyde, J. S., & Mezulis, A. H. (2020). Gender differences in depression: Biological, affective, cognitive, and sociocultural factors. *Harvard Review of Psychiatry*, *28*(1), 4–13. 31913978 10.1097/HRP.0000000000000230

[CR26] Hyde, J. S., Mezulis, A. H., & Abramson, L. Y. (2008). The ABCs of depression: Integrating affective, biological, and cognitive models to explain the emergence of the gender difference in depression. *Psychological Review*, *115*(2), 291–313. 18426291 10.1037/0033-295X.115.2.291

[CR27] Khng, K. H., & Lee, K. (2014). The relationship between Stroop and stop-signal measures of inhibition in adolescents: Influences from variations in context and measure estimation. *Plos One*, *9*(7), e101356. 24992683 10.1371/journal.pone.0101356 PMC4081588

[CR28] Krause, E. D., Vélez, C. E., Woo, R., Hoffmann, B., Freres, D. R., Abenavoli, R. M., & Gillham, J. E. (2018). Rumination, depression, and gender in early adolescence: A longitudinal study of a bidirectional model. *The Journal of Early Adolescence*, *38*(7), 923–946.

[CR29] Ladouceur, C. D., Kerestes, R., Schlund, M. W., Shirtcliff, E. A., Lee, Y., & Dahl, R. E. (2019). Neural systems underlying reward cue processing in early adolescence: The role of puberty and pubertal hormones. *Psychoneuroendocrinology*, *102*, 281–291. 30639923 10.1016/j.psyneuen.2018.12.016 PMC7085287

[CR30] Lawlor, V. M., Webb, C. A., Wiecki, T. V., Frank, M. J., Trivedi, M., Pizzagalli, D. A., & Dillon, D. G. (2020). Dissecting the impact of depression on decision-making. *Psychological medicine*, *50*(10), 1613–1622. 31280757 10.1017/S0033291719001570 PMC6946886

[CR88] Lerche, V., Voss, A., & Nagler, M. (2017). How many trials are required for parameter estimation in diffusion modeling? A comparison of different optimization criteria. *Behavior research methods*, *49*(2), 513–537. 27287445 10.3758/s13428-016-0740-2

[CR31] Lüdecke, D. (2024). _sjPlot: Data Visualization for Statistics in Social Science_. R package version 2.8.17. https://CRAN.R-project.org/package=sjPlot

[CR32] Maalouf, F. T., & Brent, D. A. (2012). Child and adolescent depression intervention overview: what works, for whom and how well? *Child and Adolescent Psychiatric Clinics*, *21*(2), 299–312. 10.1016/j.chc.2012.01.001 22537728

[CR33] Makowski, D., Ben-Shachar, M. S., & Lüdecke, D. (2019). bayestestR: Describing effects and their uncertainty, existence and significance within the Bayesian framework. *Journal of open source software*, *4*(40), 1541.

[CR34] McGuire, T. C., McCormick, K. C., Koch, M. K., & Mendle, J. (2019). Pubertal maturation and trajectories of depression during early adolescence. *Frontiers in Psychology*, *10*, 1362. 31244742 10.3389/fpsyg.2019.01362 PMC6582206

[CR35] Meinzer, M. C., Pettit, J. W., & Viswesvaran, C. (2014). The co-occurrence of attention-deficit/hyperactivity disorder and unipolar depression in children and adolescents: A meta-analytic review. *Clinical Psychology Review*, *34*(8), 595–607. 25455624 10.1016/j.cpr.2014.10.002

[CR38] Mendle, J., Beltz, A. M., Carter, R., & Dorn, L. D. (2019). *Journal of Research on Adolescence*, 29(4), 982–1001. 10.1111/jora.12371 30869839

[CR37] Mendle, J., Beam, C. R., McKone, K. M., & Koch, M. K. (2020). Puberty and transdiagnostic risks for mental health. *Journal of Research on Adolescence*, *30*(3), 687–705. 32109337 10.1111/jora.12552

[CR39] Millisecond Software (2016). Inquisit 5 [Computer software]. Retrieved from https://www.millisecond.com

[CR40] Mueller, S. C., Unal, C., Saretta, M., Al Mughairbi, F., Gómez-Odriozola, J., Calvete, E., & Metin, B. (2021). Working memory and emotional interpretation bias in a sample of Syrian refugee adolescents. *European child & adolescent psychiatry*, *30*(12), 1885–1894. 33025075 10.1007/s00787-020-01656-8

[CR41] Myers, C. E., Interian, A., & Moustafa, A. A. (2022). A practical introduction to using the drift diffusion model of decision-making in cognitive psychology, neuroscience, and health sciences. *Frontiers in Psychology*, *13*, 1039172. 36571016 10.3389/fpsyg.2022.1039172 PMC9784241

[CR42] Oppenheimer, C. W., Hankin, B. L., & Young, J. (2018). Effect of parenting and peer stressors on cognitive vulnerability and risk for depression among youth. *Journal of abnormal child psychology*, *46*(3), 597–612. 28623624 10.1007/s10802-017-0315-4 PMC5732886

[CR43] Pe, M. L., Vandekerckhove, J., & Kuppens, P. (2013). A diffusion model account of the relationship between the emotional flanker task and rumination and depression. *Emotion*, *13*(4), 739. 23527499 10.1037/a0031628

[CR44] Peckham, A. D., & Johnson, S. L. (2018). Cognitive control training for emotion-related impulsivity. *Behaviour Research and Therapy*, *105*, 17–26. 29609103 10.1016/j.brat.2018.03.009 PMC5937944

[CR45] Pedersen, M. L., Alnæs, D., van der Meer, D., Fernandez-Cabello, S., Berthet, P., Dahl, A., & Westlye, L. T. (2023). Computational modeling of the N-Back task in the ABCD study: associations of drift diffusion model parameters to polygenic scores of mental disorders and cardiometabolic diseases. *Biological Psychiatry: Cognitive Neuroscience and Neuroimaging*, *8*(3), 290–299. 35427796 10.1016/j.bpsc.2022.03.012

[CR47] Petersen, A. C., Crockett, L., Richards, M., & Boxer, A. (1988). *Journal of Youth and Adolescence*, 17(2), 117–133. 24277579 10.1007/BF01537962

[CR48] Pfeifer, J. H., & Allen, N. B. (2021). Puberty initiates cascading relationships between neurodevelopmental, social, and internalizing processes across adolescence. *Biological psychiatry*, *89*(2), 99–108. 33334434 10.1016/j.biopsych.2020.09.002 PMC8494463

[CR49] Pitliya, R. J., Nelson, B. D., Hajcak, G., & Jin, J. (2022). Drift-diffusion model reveals impaired reward-based perceptual decision-making processes associated with depression in late childhood and early adolescent girls. *Research on Child and Adolescent Psychopathology*, *50*(11), 1515–1528. 35678933 10.1007/s10802-022-00936-y

[CR51] R Core Team (2021). *R: A language and environment for statistical computing*. R Foundation for Statistical Computing, Vienna, Austria. URL https://www.R-project.org/

[CR52] Radloff, L. S. (1977). *Applied Psychological Measurement*, 1(3), 385–401.

[CR53] Ratcliff, R. (1993). Methods for dealing with reaction time outliers. *Psychological bulletin*, *114*(3), 510. 8272468 10.1037/0033-2909.114.3.510

[CR85] Ravindranath, O. (2024). The Contributions of Pubertal Maturation to the Neurobiological Mechanisms of Cognitive and Affective Development (Doctoral dissertation, University of Pittsburgh).

[CR54] Revelle, W. (2025). Psych: procedures for psychological, psychometric, and personality research (version 2.6.3). Northwestern University. https://personality-project.org/r/psych/

[CR55] Rifkin, L. S., Giollabhui, M., Kendall, N., Abramson, P. C., L. Y., & Alloy, L. B. (2021). Attention, rumination and depression in youth with negative inferential styles: A prospective study. *Journal of Affective Disorders*, *291*, 209–217. 34049190 10.1016/j.jad.2021.04.095 PMC8444224

[CR56] Schäfer, J. Ö., Naumann, E., Holmes, E. A., Tuschen-Caffier, B., & Samson, A. C. (2017). Emotion regulation strategies in depressive and anxiety symptoms in youth: A meta-analytic review. *Journal of Youth and Adolescence*, *46*, 261–276. 27734198 10.1007/s10964-016-0585-0

[CR57] Schulz, K. M., Molenda-Figueira, H. A., & Sisk, C. L. (2009). Back to the future: The organizational–activational hypothesis adapted to puberty and adolescence. *Hormones and Behavior*, *55*(5), 597–604. 19446076 10.1016/j.yhbeh.2009.03.010 PMC2720102

[CR89] Schuch, S., & Konrad, K. (2017). Investigating task inhibition in children versus adults: A diffusion model analysis. *Journal of Experimental Child Psychology*, *156*, 143–167. 28068551 10.1016/j.jecp.2016.11.012

[CR58] Servant, M., White, C., Montagnini, A., & Burle, B. (2015). Using covert response activation to test latent assumptions of formal decision-making models in humans. *Journal of Neuroscience*, *35*(28), 10371–10385. 26180211 10.1523/JNEUROSCI.0078-15.2015 PMC6605344

[CR59] Shapero, B. G., Stange, J. P., McArthur, B. A., Abramson, L. Y., & Alloy, L. B. (2019). Cognitive reappraisal attenuates the association between depressive symptoms and emotional response to stress during adolescence. *Cognition and Emotion*, *33*(3), 524–535. 29637806 10.1080/02699931.2018.1462148 PMC6185799

[CR60] Shirtcliff, E. A., Dahl, R. E., & Pollak, S. D. (2009). Pubertal development: correspondence between hormonal and physical development. *Child development*, *80*(2), 327–337. 10.1111/j.1467-8624.2009.01263.x PMC2727719 19466995

[CR61] Singmann, H., Brown, S., Gretton, M., Heathcote, A., Voss, A., Voss, J., & Terry, A. (2018, June). *Package ‘rtdists’*.

[CR62] Smid, S. C., McNeish, D., Miočević, M., & van de Schoot, R. (2020). Bayesian versus frequentist estimation for structural equation models in small sample contexts: A systematic review. *Structural Equation Modeling: A Multidisciplinary Journal*, *27*(1), 131–161.

[CR63] Sripada, C., & Weigard, A. (2021). Impaired evidence accumulation as a transdiagnostic vulnerability factor in psychopathology. *Frontiers in psychiatry*, *12*, 627179. 33679485 10.3389/fpsyt.2021.627179 PMC7925621

[CR64] Stange, J. P., Bessette, K. L., Jenkins, L. M., Peters, A. T., Feldhaus, C., Crane, N. A., Ajilore, O., Jacobs, R. H., Watkins, E. R., & Langenecker, S. A. (2017). Attenuated intrinsic connectivity within cognitive control network among individuals with remitted depression: Temporal stability and association with negative cognitive styles. *Human Brain Mapping*, *38*(6), 2939–2954. 28345197 10.1002/hbm.23564 PMC5426983

[CR83] Stumper, A., & Alloy, L. B. (2023). Associations between pubertal stage and depression: A systematic review of the literature. *Child Psychiatry & Human Development*, *54*(2), 312–339. 34529199 10.1007/s10578-021-01244-0 PMC11869324

[CR65] Stumper, A., Mac Giollabhui, N., Abramson, L. Y., & Alloy, L. B. (2020). Early pubertal timing mediates the association between low socioeconomic status and poor attention and executive functioning in a diverse community sample of adolescents. *Journal of youth and adolescence*, *49*(7), 1420–1432. 32020488 10.1007/s10964-020-01198-x PMC7302968

[CR66] Swilley-Martinez, M. E., Coles, S. A., Miller, V. E., Alam, I. Z., Fitch, K. V., Cruz, T. H., & Ranapurwala, S. I. (2023). We adjusted for race: now what? A systematic review of utilization and reporting of race in American Journal of Epidemiology and Epidemiology, 2020–2021. *Epidemiologic reviews*, *45*(1), 15–31. 37789703 10.1093/epirev/mxad010 PMC12098948

[CR67] Tomlinson, R. C., Weigard, A. S., Sripada, C., Jonides, J., Klump, K. L., Burt, S. A., & Hyde, L. W. (2025). Efficiency of evidence accumulation as a formal model-based measure of task-general executive functioning in adolescents. *Journal of Psychopathology and Clinical Science*. 10.1037/abn0001043 PMC12373008 40839478

[CR68] Tukey, J. W. (1977). *Exploratory data analysis* (Vol. 2, pp. 131–160). Addison-wesley.

[CR69] Turvey, C. L., Wallace, R. B., & Herzog, R. (1999). *International Psychogeriatrics*, 11(2), 139–148. (Introduces/validates the 8-item CES-D.). 11475428 10.1017/s1041610299005694

[CR70] Ullsperger, J. M., & Nikolas, M. A. (2017). A meta-analytic review of the association between pubertal timing and psychopathology in adolescence: Are there sex differences in risk? *Psychological Bulletin*, *143*(9), 903–938. 28530427 10.1037/bul0000106

[CR71] Vijayakumar, N., Whittle, S., Yücel, M., Byrne, M. L., Schwartz, O., Simmons, J. G., & Allen, N. B. (2016). Impaired maturation of cognitive control in adolescents who develop major depressive disorder. *Journal of Clinical Child & Adolescent Psychology*, *45*(1), 31–43. 25700138 10.1080/15374416.2014.987381

[CR72] Wagenmakers, E. J., Van Der Maas, H. L., & Grasman, R. P. (2007). An EZ-diffusion model for response time and accuracy. *Psychonomic bulletin & review*, *14*(1), 3–22. 17546727 10.3758/bf03194023

[CR73] Wagner, S., Müller, C., Helmreich, I., Huss, M., & Tadić, A. (2015). A meta-analysis of cognitive functions in children and adolescents with major depressive disorder. *European Child & Adolescent Psychiatry*, *24*, 5–19. 24869711 10.1007/s00787-014-0559-2

[CR87] Warren, S. L., Malaiya, R. K., Drake, O. K., Tang, A., & Chandra, N. K. (2025). Cognitive Control Decision-Making Dynamics Across Internalizing and Externalizing Symptoms in Youth. *Research on Child and Adolescent Psychopathology*, *53*(10), 1539–1554. 40699480 10.1007/s10802-025-01351-9

[CR75] Weigard, A., & Sripada, C. (2021). Task-general efficiency of evidence accumulation as a computationally defined neurocognitive trait: Implications for clinical neuroscience. *Biological psychiatry global open science*, *1*(1), 5–15. 35317408 10.1016/j.bpsgos.2021.02.001 PMC8936715

[CR76] White, C. N., Ratcliff, R., Vasey, M. W., & McKoon, G. (2010). Using diffusion models to understand clinical disorders. *Journal of mathematical psychology*, *54*(1), 39–52. 20431690 10.1016/j.jmp.2010.01.004 PMC2859713

[CR77] White, N., Kouwenhoven, M., & Machado, L. (2022). Short-term retest performance in young versus older adults: Consideration of integrated speed-accuracy measures. *Experimental aging research*, *48*(1), 68–85. 33993852 10.1080/0361073X.2021.1919475

[CR78] Wickham, H., Averick, M., Bryan, J., Chang, W., McGowan, L. D., François, R., Grolemund, G., Hayes, A., Henry, L., Hester, J., Kuhn, M., Pedersen, T. L., Miller, E., Bache, S. M., Müller, K., Ooms, J., Robinson, D., Seidel, D. P., Spinu, V., & Yutani, H. (2019). Welcome to the tidyverse. *Journal of Open Source Software*, *4*(43), 1686. 10.21105/joss.01686

[CR79] Wickham, H., François, R., Henry, L., Müller, K., & Vaughan, D. (2023). dplyr: A grammar of data manipulation (Version 1.1.4) [R package]. https://CRAN.R-project.org/package=dplyr

[CR80] Williams, J. M. G., Mathews, A., & MacLeod, C. (1996). *Psychological Bulletin*, 120(1), 3–24. 8711015 10.1037/0033-2909.120.1.3

[CR81] Wilson, S., & Dumornay, N. M. (2022). Rising rates of adolescent depression in the United States: Challenges and opportunities in the 2020s. *Journal of Adolescent Health*, *70*(3), 354–355. 10.1016/j.jadohealth.2021.12.003 PMC8868033 35183317

[CR82] Xiang, Y., Cao, R., & Li, X. (2024). Parental education level and adolescent depression: A multi-country meta-analysis. *Journal of Affective Disorders*, *347*, 645–655. 38008290 10.1016/j.jad.2023.11.081

