# Supplementary material for: Examining Pubertal and Cognitive Differences in Adolescent Depressive Symptoms Using Drift Diffusion Modeling
Source: Res Child Adolesc Psychopathol. 2026 Aug 14;54(4):99. doi: 10.1007/s10802-026-01493-4 (PMC13476296; doi:10.1007/s10802-026-01493-4)
Supplement: Supplementary file 1 [file 10802_2026_1493_MOESM1_ESM.docx]

**Table S1.** Moderation Analyses of Pubertal Timing x Drift Rate on Depressive Symptoms

|  | Stroop | | | | Emotional Stroop | | | |
| --- | --- | --- | --- | --- | --- | --- | --- | --- |
|  | Main Effects | | Moderation | | Main Effects | | Moderation | |
| *Coefficient* | *Estimates* | *CI* | *Estimates* | *CI* | *Estimates* | *CI* | *Estimates* | *CI* |
| Intercept | 0.57 | -0.12 – 1.26 | 0.52 | -0.19 – 1.22 | -0.07 | -0.79 – 0.66 | -0.08 | -0.82 – 0.65 |
| Male | -0.18 ^*^ | -0.34 – -0.01 | -0.17 ^*^ | -0.34 – -0.00 | -0.14 | -0.30 – 0.03 | -0.13 | -0.30 – 0.04 |
| Age | -0.01 | -0.05 – 0.03 | -0.01 | -0.05 – 0.03 | 0.00 | -0.04 – 0.04 | 0.00 | -0.04 – 0.04 |
| Income to needs | 0.04 | -0.03 – 0.11 | 0.04 | -0.03 – 0.12 | 0.04 | -0.03 – 0.12 | 0.05 | -0.03 – 0.12 |
| College graduate | -0.20 | -0.39 – 0.00 | -0.19 | -0.39 – 0.01 | -0.22 ^*^ | -0.41 – -0.03 | -0.23 ^*^ | -0.43 – -0.04 |
| Black race/ethnicity | 0.11 | -0.11 – 0.33 | 0.12 | -0.10 – 0.34 | 0.15 | -0.07 – 0.38 | 0.15 | -0.07 – 0.37 |
| Hispanic, Latino or Spanish race/ethnicity | 0.02 | -0.19 – 0.24 | 0.02 | -0.19 – 0.24 | 0.02 | -0.20 – 0.23 | 0.02 | -0.20 – 0.23 |
| American Indian or Alaskan Native | 0.49 | -0.03 – 1.01 | 0.47 | -0.06 – 0.99 | 0.51 ^*^ | 0.01 – 1.01 | 0.52 ^*^ | 0.02 – 1.03 |
| Asian race/ethnicity | 0.30 | -0.02 – 0.61 | 0.29 | -0.02 – 0.61 | 0.32 ^*^ | 0.02 – 0.63 | 0.33 ^*^ | 0.02 – 0.64 |
| Pubertal timing | 0.13 ^*^ | 0.01 – 0.25 | 0.31 | -0.14 – 0.76 | 0.13 ^*^ | 0.01 – 0.25 | 0.33 | -0.38 – 1.04 |
| Drift rate | -0.03 | -0.15 – 0.09 | -0.02 | -0.14 – 0.10 | 0.22 | -0.11 – 0.55 | 0.23 | -0.10 – 0.56 |
| Pubertal timing x Drift rate |  |  | -.08 | -.27 - .11 |  |  | -0.12 | -0.56 – 0.31 |
| Observations | 99 | | 95 | | 99 | | 95 | |
| R^2^ / R^2^ adjusted | 0.178 / 0.085 | | 0.214 / 0.120 | | 0.184 / 0.081 | | 0.217 / 0.113 | |
| ** p<0.05   ** p<0.01   *** p<0.001 Note:* Mother education was collapsed into 0= Less than college degree, 1 = college degree or higher; Pubertal timing, positive values indicate earlier pubertal timing, negative values indicate later pubertal timing. | | | | | | | | |

**Table S2.** Moderation Analyses of Pubertal Timing x Non-Decision Time on Depressive Symptoms

|  | Stroop | | | | Emotional Stroop | | | |
| --- | --- | --- | --- | --- | --- | --- | --- | --- |
|  | Main Effects | | Moderation | | Main Effects | | Moderation | |
| *Coefficient* | *Estimates*  ($\beta$) | *CI* | *Estimates*  ($\beta$) | *CI* | *Estimates*  ($\beta$) | *CI* | *Estimates*  ($\beta$) | *CI* |
| Intercept | 0.32 | -0.24 – 0.88 | 0.32 | -0.25 – 0.88 | 1.15 ^**^ | 0.32 – 1.99 | 1.14 ^**^ | 0.28 – 2.00 |
| Male | -0.18 ^*^ | -0.34 – -0.01 | -0.17 ^*^ | -0.34 – -0.01 | -0.19 ^*^ | -0.36 – -0.03 | -0.19 ^*^ | -0.36 – -0.03 |
| Age | -0.00 | -0.04 – 0.04 | -0.00 | -0.04 – 0.04 | -0.02 | -0.07 – 0.02 | -0.02 | -0.07 – 0.02 |
| Income to needs | 0.04 | -0.03 – 0.12 | 0.04 | -0.03 – 0.12 | 0.03 | -0.04 – 0.10 | 0.03 | -0.04 – 0.10 |
| College graduate | -0.20 ^*^ | -0.40 – -0.01 | -0.21 ^*^ | -0.40 – -0.01 | -0.18 | -0.37 – 0.00 | -0.18 | -0.37 – 0.00 |
| Black race/ethnicity | 0.06 | -0.16 – 0.29 | 0.06 | -0.16 – 0.29 | 0.14 | -0.07 – 0.35 | 0.14 | -0.07 – 0.36 |
| Hispanic, Latino or Spanish race/ethnicity | 0.00 | -0.21 – 0.22 | -0.01 | -0.22 – 0.21 | 0.06 | -0.15 – 0.27 | 0.06 | -0.15 – 0.27 |
| American Indian or Alaskan Native | 0.43 | -0.07 – 0.93 | 0.43 | -0.07 – 0.94 | 0.52 ^*^ | 0.04 – 1.00 | 0.52 ^*^ | 0.04 – 1.01 |
| Asian race/ethnicity | 0.26 | -0.05 – 0.57 | 0.26 | -0.06 – 0.57 | 0.27 | -0.02 – 0.57 | 0.28 | -0.03 – 0.58 |
| Pubertal timing | 0.13 ^*^ | 0.01 – 0.25 | 0.20 | -0.04 – 0.45 | 0.13 ^*^ | 0.02 – 0.25 | 0.10 | -0.35 – 0.55 |
| Non-decision time | 0.17 | -0.06 – 0.40 | 0.18 | -0.05 – 0.41 | -0.68 ^**^ | -1.15 – -0.20 | -0.67 ^**^ | -1.16 – -0.18 |
| Pubertal timing x non-decision time |  |  | -0.15 | -0.55 – 0.26 |  |  | 0.04 | -0.55 – 0.64 |
| Observations | 99 | | 99 | | 95 | | 95 | |
| R^2^ / R^2^ adjusted | 0.196 / 0.105 | | 0.201 / 0.100 | | 0.268 / 0.180 | | 0.268 / 0.171 | |
| ** p<0.05   ** p<0.01   *** p<0.001*  *Note:* Mother education was collapsed into 0= Less than college degree, 1 = college degree or higher; Pubertal timing, positive values indicate earlier pubertal timing, negative values indicate later pubertal timing*.* | | | | | | | | |

**Table S3.** Three-way Interaction Among Sex, Pubertal Timing, and Boundary Separation on Depressive Symptoms

|  | Stroop  Main Effects | | Stroop  Moderation | | Emotional Stroop  Main Effects | | Emotional Stroop Moderation | |
| --- | --- | --- | --- | --- | --- | --- | --- | --- |
| *Coefficient* | *Estimates* | *CI* | *Estimates* | *CI* | *Estimates* | *CI* | *Estimates* | *CI* |
| Intercept | 0.29 | -0.44 – 1.03 | 0.29 | -0.44 – 1.03 | -0.26 | -1.01 – 0.49 | -0.23 | -0.97 – 0.52 |
| Male | -0.14 | -0.32 – 0.03 | -0.14 | -0.31 – 0.04 | -0.11 | -0.28 – 0.06 | -0.10 | -0.27 – 0.07 |
| Age | -0.01 | -0.05 – 0.03 | -0.01 | -0.05 – 0.03 | -0.00 | -0.04 – 0.04 | -0.01 | -0.05 – 0.03 |
| Parent depression | 0.20 | -0.01 – 0.41 | 0.19 | -0.02 – 0.40 | 0.15 | -0.06 – 0.36 | 0.14 | -0.07 – 0.34 |
| Income to needs | 0.06 | -0.02 – 0.14 | 0.06 | -0.02 – 0.13 | 0.06 | -0.02 – 0.14 | 0.06 | -0.02 – 0.14 |
| College graduate | -0.21 ^*^ | -0.41 – -0.01 | -0.21 ^*^ | -0.41 – -0.01 | -0.24 ^*^ | -0.44 – -0.04 | -0.24 ^*^ | -0.43 – -0.05 |
| Black race/ethnicity | 0.12 | -0.10 – 0.34 | 0.13 | -0.09 – 0.36 | 0.15 | -0.07 – 0.38 | 0.19 | -0.04 – 0.41 |
| Hispanic, Latino or Spanish race/ethnicity | -0.02 | -0.24 – 0.20 | 0.00 | -0.22 – 0.22 | -0.01 | -0.23 – 0.20 | 0.02 | -0.20 – 0.23 |
| American Indian or Alaskan Native | 0.45 | -0.07 – 0.98 | 0.46 | -0.06 – 0.99 | 0.54 ^*^ | 0.03 – 1.04 | 0.56 ^*^ | 0.06 – 1.05 |
| Asian race/ethnicity | 0.35 ^*^ | 0.02 – 0.69 | 0.33 | -0.00 – 0.67 | 0.38 ^*^ | 0.05 – 0.70 | 0.33 ^*^ | 0.01 – 0.66 |
| Pubertal timing | 0.34 | -0.11 – 0.79 | 0.32 | -0.13 – 0.77 | 0.27 | -0.45 – 0.99 | 0.33 | -0.38 – 1.04 |
| Stroop boundary separation | 0.00 | -0.12 – 0.12 | 0.00 | -0.12 – 0.12 |  |  |  |  |
| Pubertal timing x Stroop boundary separation | -0.10 | -0.29 – 0.09 | -0.05 | -0.26 – 0.17 |  |  |  |  |
| Male x pubertal timing x Stroop boundary separation |  |  | -0.06 | -0.17 – 0.06 |  |  |  |  |
| Emotional Stroop boundary separation |  |  |  |  | 0.30 | -0.04 – 0.63 | 0.27 | -0.06 – 0.61 |
| Pubertal timing x emotional Stroop boundary separation |  |  |  |  | -0.10 | -0.54 – 0.34 | -0.04 | -0.47 – 0.40 |
| Male x pubertal timing x emotional Stroop boundary separation |  |  |  |  |  |  | -0.15 | -0.31 – 0.00 |
| Observations | 95 | | 95 | | 91 | | 91 | |
| R^2^ / R^2^ adjusted | 0.218 / 0.104 | | 0.228 / 0.105 | | 0.246 / 0.130 | | 0.282 / 0.161 | |
| ** p<0.05   ** p<0.01   *** p<0.001* | | | | | | | | |

*Note:* Mother education was collapsed into 0= Less than college degree, 1 = college degree or higher; Pubertal timing, positive values indicate earlier pubertal timing, negative values indicate later pubertal timing.

**Table S4.** Three-way Interaction Among Sex, Pubertal Timing, and Drift Rate on Depressive Symptoms

|  | Stroop  Main Effects | | Stroop  Moderation | | Emotional Stroop  Main Effects | | Emotional Stroop Moderation | |
| --- | --- | --- | --- | --- | --- | --- | --- | --- |
| *Coefficient* | *Estimates* | *CI* | *Estimates* | *CI* | *Estimates* | *CI* | *Estimates* | *CI* |
| Intercept | 0.39 | -0.16 – 0.94 | 0.39 | -0.15 – 0.94 | 0.07 | -0.50 – 0.63 | 0.06 | -0.49 – 0.62 |
| Male | -0.11 | -0.29 – 0.06 | -0.09 | -0.26 – 0.08 | -0.10 | -0.27 – 0.07 | -0.08 | -0.24 – 0.09 |
| Age | -0.01 | -0.05 – 0.03 | -0.01 | -0.05 – 0.02 | 0.01 | -0.03 – 0.06 | 0.01 | -0.03 – 0.05 |
| Parent depression | 0.18 | -0.02 – 0.39 | 0.17 | -0.04 – 0.37 | 0.18 | -0.03 – 0.38 | 0.17 | -0.03 – 0.38 |
| Income to needs | 0.05 | -0.03 – 0.13 | 0.04 | -0.03 – 0.12 | 0.05 | -0.03 – 0.12 | 0.04 | -0.03 – 0.11 |
| College graduate | -0.18 | -0.38 – 0.02 | -0.16 | -0.36 – 0.04 | -0.23 ^*^ | -0.42 – -0.03 | -0.22 ^*^ | -0.41 – -0.03 |
| Black race/ethnicity | 0.11 | -0.11 – 0.32 | 0.13 | -0.08 – 0.35 | 0.16 | -0.06 – 0.37 | 0.19 | -0.03 – 0.41 |
| Hispanic, Latino or Spanish race/ethnicity | -0.03 | -0.24 – 0.19 | 0.01 | -0.20 – 0.22 | -0.04 | -0.25 – 0.17 | -0.01 | -0.22 – 0.20 |
| American Indian or Alaskan Native | 0.53 ^*^ | 0.03 – 1.03 | 0.52 ^*^ | 0.03 – 1.01 | 0.54 ^*^ | 0.05 – 1.02 | 0.56 ^*^ | 0.08 – 1.04 |
| Asian race/ethnicity | 0.31 | -0.02 – 0.64 | 0.22 | -0.12 – 0.55 | 0.39 ^*^ | 0.08 – 0.70 | 0.35 ^*^ | 0.03 – 0.66 |
| Pubertal timing | -0.12 | -0.37 – 0.12 | -0.10 | -0.34 – 0.15 | -0.30 | -0.64 – 0.05 | -0.30 | -0.64 – 0.04 |
| Stroop drift rate | -0.05 | -0.21 – 0.12 | -0.07 | -0.24 – 0.10 |  |  |  |  |
| Pubertal timing x Stroop drift rate | 0.25 ^*^ | 0.03 – 0.46 | 0.35 ^**^ | 0.11 – 0.58 |  |  |  |  |
| Male x pubertal timing x Stroop drift rate |  |  | -0.22 | -0.45 – 0.00 |  |  |  |  |
| Emotional Stroop drift rate |  |  |  |  | -0.04 | -0.15 – 0.08 | -0.05 | -0.16 – 0.07 |
| Pubertal timing x emotional Stroop drift rate |  |  |  |  | 0.23 ^**^ | 0.06 – 0.40 | 0.30 ^**^ | 0.11 – 0.49 |
| Male x pubertal timing x emotional Stroop drift rate |  |  |  |  |  |  | -0.11 | -0.24 – 0.02 |
| Observations | 95 | | 95 | | 91 | | 91 | |
| R^2^ / R^2^ adjusted | 0.256 / 0.147 | | 0.290 / 0.177 | | 0.298 / 0.190 | | 0.323 / 0.208 | |
| ** p<0.05   ** p<0.01   *** p<0.001* | | | | | | | | |

Note: Mother education was collapsed into 0= Less than college degree, 1 = college degree or higher; Pubertal timing, positive values indicate earlier pubertal timing, negative values indicate later pubertal timing.

**Table S5.** Three-way Interaction Among Sex, Pubertal Timing, and Non-Decision Time on Depressive Symptoms

|  | Stroop  Main Effects | | Stroop  Moderation | | Emotional Stroop  Main Effects | | Emotional Stroop Moderation | |
| --- | --- | --- | --- | --- | --- | --- | --- | --- |
| *Coefficient* | *Estimates* | *CI* | *Estimates* | *CI* | *Estimates* | *CI* | *Estimates* | *CI* |
| Intercept | 0.22 | -0.36 – 0.80 | 0.21 | -0.38 – 0.79 | 1.04 ^*^ | 0.15 – 1.93 | 1.06 ^*^ | 0.17 – 1.95 |
| Male | -0.15 | -0.32 – 0.02 | -0.14 | -0.31 – 0.03 | -0.18 ^*^ | -0.35 – -0.00 | -0.18 ^*^ | -0.35 – -0.00 |
| Age | -0.01 | -0.04 – 0.03 | -0.01 | -0.05 – 0.03 | -0.02 | -0.07 – 0.02 | -0.02 | -0.07 – 0.02 |
| Parent depression | 0.18 | -0.03 – 0.39 | 0.18 | -0.03 – 0.39 | 0.12 | -0.08 – 0.33 | 0.12 | -0.09 – 0.33 |
| Income to needs | 0.06 | -0.02 – 0.13 | 0.06 | -0.02 – 0.13 | 0.04 | -0.04 – 0.11 | 0.04 | -0.04 – 0.11 |
| College graduate | -0.23 ^*^ | -0.43 – -0.03 | -0.23 ^*^ | -0.43 – -0.03 | -0.19 | -0.39 – 0.00 | -0.19 | -0.39 – 0.00 |
| Black race/ethnicity | 0.07 | -0.16 – 0.29 | 0.09 | -0.14 – 0.32 | 0.14 | -0.07 – 0.36 | 0.15 | -0.06 – 0.37 |
| Hispanic, Latino or Spanish race/ethnicity | -0.05 | -0.26 – 0.17 | -0.03 | -0.25 – 0.20 | 0.03 | -0.18 – 0.25 | 0.05 | -0.17 – 0.27 |
| American Indian or Alaskan Native | 0.44 | -0.07 – 0.94 | 0.45 | -0.05 – 0.96 | 0.52 ^*^ | 0.03 – 1.01 | 0.52 ^*^ | 0.03 – 1.01 |
| Asian race/ethnicity | 0.31 | -0.03 – 0.64 | 0.29 | -0.05 – 0.63 | 0.31 | -0.01 – 0.63 | 0.30 | -0.02 – 0.62 |
| Pubertal timing | 0.20 | -0.04 – 0.45 | 0.19 | -0.06 – 0.44 | 0.10 | -0.35 – 0.55 | 0.12 | -0.34 – 0.58 |
| Stroop non-decision time | 0.18 | -0.06 – 0.41 | 0.17 | -0.06 – 0.41 |  |  |  |  |
| Pubertal timing x Stroop non-decision time | -0.18 | -0.61 – 0.24 | -0.02 | -0.58 – 0.55 |  |  |  |  |
| Male x pubertal timing x Stroop non-decision time |  |  | -0.21 | -0.67 – 0.26 |  |  |  |  |
| Emotional Stroop non-decision time |  |  |  |  | -0.64 ^*^ | -1.13 – -0.15 | -0.65 ^*^ | -1.15 – -0.16 |
| Pubertal timing x emotional Stroop non-decision time |  |  |  |  | 0.03 | -0.57 – 0.62 | 0.07 | -0.54 – 0.68 |
| Male x pubertal timing x emotional Stroop non-decision time |  |  |  |  |  |  | -0.11 | -0.44 – 0.22 |
| Observations | 95 | | 95 | | 91 | | 91 | |
| R^2^ / R^2^ adjusted | 0.234 / 0.122 | | 0.241 / 0.120 | | 0.282 / 0.171 | | 0.286 / 0.166 | |
| ** p<0.05   ** p<0.01   *** p<0.001* | | | | | | | | |

Note: Mother education was collapsed into 0= Less than college degree, 1 = college degree or higher; Pubertal timing, positive values indicate earlier pubertal timing, negative values indicate later pubertal timing.

**Table S6.** Bayesian Main Effect and Moderation Analyses of Pubertal Timing x Drift Rate on Depressive Symptoms

| Stroop | | | | | | | | | | |
| --- | --- | --- | --- | --- | --- | --- | --- | --- | --- | --- |
|  | Main effects | | | | Moderation | | | 3-way moderation | | |
|  | **Median** | | **95% CI** | **PD** | **Median** | **95% CI** | **PD** | **Median** | **95% CI** | **PD** |
| (Intercept) | 0.46 | | [−0.08, 0.99] | 95.38% | 0.5 | [−0.04, 1.04] | 96.75% | 0.5 | [-0.01, 1.00] | 97.32% |
| Male | -0.17 | | [−0.34, −0.01] | 97.88% | −0.13 | [−0.30, 0.03] | 94.05% | -0.11 | [-0.28, 0.05] | 91.35% |
| Age | -9.34E-03 | | [−0.05, 0.03] | 68.27% | −0.009 | [−0.05, 0.03] | 68.73% | -0.01 | [-0.05, 0.03] | 72.45% |
| Income to needs | 0.04 | | [−0.04, 0.11] | 84.88% | 0.03 | [−0.04, 0.11] | 82.00% | 0.03 | [-0.04, 0.10] | 76.15% |
| College graduate | -0.19 | | [−0.38, 0.01] | 96.65% | −0.16 | [−0.35, 0.03] | 94.53% | -0.13 | [-0.32, 0.07] | 90.50% |
| Race/ethnicity |  | |  |  |  |  |  |  |  |  |
| Black | 0.11 | | [−0.11, 0.32] | 83.60% | 0.1 | [−0.12, 0.31] | 82.60% | 0.13 | [-0.08, 0.35] | 88.85% |
| Hispanic, Latino or Spanish | 0.02 | | [−0.19, 0.23] | 58.10% | 0.009 | [−0.20, 0.22] | 53.52% | 0.04 | [-0.17, 0.25] | 66.17% |
| American Indian or Alaskan Native | 0.45 | | [−0.05, 0.96] | 96.17% | 0.52 | [0.02, 1.02] | 97.92% | 0.49 | [ 0.02, 0.98] | 97.88% |
| Asian race/ethnicity | 0.31 | | [−0.02, 0.61] | 96.60% | 0.26 | [−0.05, 0.56] | 95.15% | 0.18 | [-0.13, 0.49] | 88.05% |
| Pubertal timing | 0.13 | | [0.01, 0.25] | 98.52% | −0.13 | [−0.36, 0.11] | 83.65% | -0.09 | [-0.32, 0.13] | 78.27% |
| Drift rate | 0.01 | | [−0.16, 0.18] | 57.35% | −0.05 | [−0.22, 0.12] | 70.93% | -0.08 | [-0.24, 0.08] | 82.40% |
| Pubertal timing *x* drift rate | | |  |  | 0.26 | [0.05, 0.47] | 99.25% | 0.37 | [ 0.16, 0.59] | 99.92% |
| Male *x* pubertal timing *x* drift rate | | | |  |  |  |  | -0.26 | [-0.47, -0.05] | 99.28% |
| Emotional Stroop | | | | | | | | | | |
|  | | Main effects | | | Moderation | | | 3-way moderation | | |
|  | | **Median** | **95% CI** | **PD** | **Median** | **95% CI** | **PD** | **Median** | **95% CI** | **PD** |
| (Intercept) | | 0.21 | [−0.32, 0.78] | 76.20% | 0.19 | [−0.35, 0.72] | 75.65% | 0.2 | [-0.33, 0.70] | 77.95% |
| Male | | −0.15 | [−0.32, 0.02] | 96.25% | −0.13 | [−0.29, 0.03] | 93.80% | -0.11 | [-0.28, 0.05] | 91.60% |
| Age | | 0.02 | [−0.03, 0.06] | 81.62% | 0.01 | [−0.03, 0.06] | 72.38% | 0.01 | [-0.03, 0.05] | 70.10% |
| Income to needs | | 0.04 | [−0.03, 0.11] | 83.88% | 0.03 | [−0.04, 0.10] | 80.17% | 0.02 | [-0.04, 0.09] | 75.30% |
| College graduate | | −0.20 | [−0.40, −0.02] | 98.17% | −0.21 | [−0.40, −0.03] | 99.02% | -0.2 | [-0.38, -0.02] | 98.45% |
| Race/ethnicity | |  |  |  |  |  |  |  |  |  |
| Black | | 0.13 | [−0.09, 0.36] | 87.05% | 0.16 | [−0.06, 0.38] | 92.07% | 0.19 | [-0.02, 0.41] | 95.83% |
| Hispanic, Latino or Spanish | | −0.009 | [−0.22, 0.20] | 53.25% | −0.02 | [−0.23, 0.19] | 57.25% | 5.68E-03 | [-0.20, 0.22] | 52.40% |
| American Indian or Alaskan Native | | 0.47 | [−0.04, 0.96] | 96.78% | 0.52 | [0.02, 1.00] | 98.12% | 0.51 | [ 0.05, 1.01] | 98.62% |
| Asian race/ethnicity | | 0.31 | [0.01, 0.62] | 97.92% | 0.35 | [0.06, 0.65] | 99.12% | 0.33 | [ 0.04, 0.63] | 98.55% |
| Pubertal timing | | 0.15 | [0.02, 0.27] | 98.88% | −0.30 | [−0.62, 0.04] | 95.65% | -0.27 | [-0.60, 0.05] | 95.47% |
| Drift rate | | −0.07 | [−0.18, 0.04] | 90.25% | −0.03 | [−0.14, 0.09] | 70.08% | -0.04 | [-0.15, 0.07] | 76.30% |
| Pubertal timing *x* drift rate | | |  |  | 0.23 | [0.06, 0.39] | 99.72% | 0.29 | [ 0.12, 0.46] | 99.90% |
| Male *x* pubertal timing *x* drift rate | | | |  |  |  |  | -0.11 | [-0.23, 0.01] | 96.43% |

*Note:* Values are Bayesian posterior medians with 95% credible intervals. PD = probability of direction; ROPE = region of practical equivalence, defined as [−0.04, 0.04]; % in ROPE = percentage of the posterior distribution within the ROPE; ESS = effective sample size.

**Table S7.** Bayesian Main Effect and Moderation Analyses of Pubertal Timing x Non-Decision on Depressive Symptoms

| Stroop | | | | | | | | | |
| --- | --- | --- | --- | --- | --- | --- | --- | --- | --- |
|  | Main Effects | | | Moderation | | | 3-Way Moderation | | |
|  | **Median** | **95% CI** | **PD** | **Median** | **95% CI** | **PD** | **Median** | **95% CI** | **PD** |
| (Intercept) | 0.32 | [-0.24, 0.88] | 86.78% | 0.32 | [-0.23, 0.86] | 87.05% | 0.3 | [-0.25, 0.85] | 85.30% |
| Male | -0.18 | [-0.34, 0.00] | 97.70% | -0.17 | [-0.34, 0.00] | 97.58% | -0.16 | [-0.32, 0.00] | 97.35% |
| Age | -3.21E-03 | [-0.04, 0.04] | 55.95% | -2.72E-03 | [-0.04, 0.04] | 55.73% | -0.00274 | [-0.04, 0.04] | 55.75% |
| Income to needs | 0.04 | [-0.03, 0.12] | 88.05% | 0.04 | [-0.02, 0.11] | 88.20% | 0.04 | [-0.04, 0.11] | 84.28% |
| College graduate | -0.21 | [-0.39, -0.01] | 98.15% | -0.2 | [-0.40, -0.02] | 98.38% | -0.21 | [-0.39, -0.01] | 97.82% |
| Race/ethnicity |  |  |  |  |  |  |  |  |  |
| Black | 0.07 | [-0.17, 0.29] | 70.43% | 0.06 | [-0.17, 0.28] | 70.55% | 0.09 | [-0.14, 0.32] | 78.35% |
| Hispanic, Latino or Spanish | 6.53E-03 | [-0.21, 0.22] | 52.08% | -6.52E-03 | [-0.22, 0.21] | 52.48% | 0.01 | [-0.20, 0.23] | 54.55% |
| American Indian or Alaskan Native | 0.43 | [-0.06, 0.95] | 95.53% | 0.44 | [-0.06, 0.91] | 96.15% | 0.44 | [-0.07, 0.95] | 95.70% |
| Asian race/ethnicity | 0.26 | [-0.06, 0.59] | 94.60% | 0.26 | [-0.06, 0.59] | 94.85% | 0.24 | [-0.07, 0.56] | 93.23% |
| Pubertal timing | 0.13 | [ 0.01, 0.25] | 98.15% | 0.2 | [-0.04, 0.44] | 95.23% | 0.2 | [-0.05, 0.44] | 94.20% |
| Non-decision time | 0.18 | [-0.06, 0.39] | 92.80% | 0.18 | [-0.04, 0.40] | 94.35% | 0.18 | [-0.05, 0.41] | 93.95% |
| Pubertal timing *x n*on-decision time |  |  |  | -0.14 | [-0.53, 0.25] | 76.40% | 0.06 | [-0.41, 0.56] | 60.10% |
| Male *x* pubertal timing *x n*on-decision time | |  |  |  |  |  | -0.3 | [-0.71, 0.10] | 93.17% |
| Emotional Stroop | | | | | | | | | |
|  | Main Effects | | | Moderation | | | 3-Way Moderation | | |
|  | **Median** | **95% CI** | **PD** | **Median** | **95% CI** | **PD** | **Median** | **95% CI** | **PD** |
| (Intercept) | 1.15 | [ 0.29, 2.01] | 99.58% | 1.14 | [ 0.29, 1.98] | 99.78% | 1.18 | [ 0.33, 1.99] | 99.58% |
| Male | -0.19 | [-0.36, -0.03] | 98.85% | -0.19 | [-0.36, -0.03] | 98.95% | -0.2 | [-0.36, -0.02] | 98.80% |
| Age | -0.02 | [-0.07, 0.02] | 83.88% | -0.02 | [-0.07, 0.02] | 84.12% | -0.02 | [-0.07, 0.02] | 86.88% |
| Income to needs | 0.03 | [-0.04, 0.10] | 79.27% | 0.03 | [-0.04, 0.10] | 77.65% | 0.02 | [-0.04, 0.09] | 76.15% |
| College graduate | -0.18 | [-0.37, 0.00] | 97.40% | -0.18 | [-0.37, 0.00] | 97.12% | -0.18 | [-0.36, 0.00] | 97.38% |
| Race/ethnicity |  |  |  |  |  |  |  |  |  |
| Black | 0.14 | [-0.07, 0.35] | 90.60% | 0.14 | [-0.08, 0.36] | 89.12% | 0.16 | [-0.06, 0.37] | 91.97% |
| Hispanic, Latino or Spanish | 0.06 | [-0.15, 0.27] | 71.70% | 0.06 | [-0.15, 0.28] | 71.28% | 0.08 | [-0.13, 0.28] | 77.00% |
| American Indian or Alaskan Native | 0.52 | [ 0.05, 0.99] | 98.50% | 0.53 | [ 0.04, 1.02] | 98.28% | 0.53 | [ 0.03, 1.01] | 98.00% |
| Asian race/ethnicity | 0.27 | [-0.03, 0.57] | 96.20% | 0.27 | [-0.03, 0.57] | 96.45% | 0.27 | [-0.03, 0.57] | 96.03% |
| Pubertal timing | 0.13 | [ 0.01, 0.26] | 98.50% | 0.1 | [-0.35, 0.55] | 66.72% | 0.13 | [-0.33, 0.56] | 71.47% |
| Non-decision time | -0.67 | [-1.13, -0.19] | 99.78% | -0.66 | [-1.16, -0.17] | 99.75% | -0.68 | [-1.17, -0.21] | 99.78% |
| Pubertal timing *x n*on-decision time |  |  |  | 0.05 | [-0.54, 0.64] | 56.75% | 0.12 | [-0.46, 0.72] | 66.45% |
| Male *x* pubertal timing *x n*on-decision time | |  |  |  |  |  | -0.18 | [-0.50, 0.12] | 88.45% |

*Note*: Values are Bayesian posterior medians with 95% credible intervals. PD = probability of direction; ROPE = region of practical equivalence, defined as [−0.04, 0.04]; % in ROPE = percentage of the posterior distribution within the ROPE; ESS = effective sample size.

**Table S8.** Bayesian Main Effect and Moderation Analyses of Pubertal Timing x Boundary Separation on Depressive Symptoms

| Stroop | | | | | | | | | | |
| --- | --- | --- | --- | --- | --- | --- | --- | --- | --- | --- |
|  | Main Effects | | | | Moderation | | | 3-Way Moderation | | |
|  | **Median** | | **95% CI** | **PD** | **Median** | **95% CI** | **PD** | **Median** | **95% CI** | **PD** |
| (Intercept) | 0.57 | | [-0.12, 1.29] | 94.42% | 0.52 | [-0.17, 1.24] | 93.20% | 0.5 | [-0.20, 1.18] | 91.90% |
| Male | -0.17 | | [-0.34, -0.01] | 97.75% | -0.17 | [-0.33, -0.01] | 98.15% | -0.17 | [-0.33, -0.01] | 98.05% |
| Age | -0.01 | | [-0.05, 0.03] | 70.93% | -9.75E-03 | [-0.05, 0.03] | 67.95% | -0.01 | [-0.05, 0.03] | 68.97% |
| Income to needs | 0.04 | | [-0.04, 0.12] | 85.40% | 0.04 | [-0.03, 0.11] | 85.78% | 0.04 | [-0.04, 0.11] | 83.90% |
| College graduate | -0.19 | | [-0.39, 0.01] | 97.00% | -0.19 | [-0.39, 0.01] | 97.05% | -0.19 | [-0.39, 0.01] | 96.80% |
| Race/ethnicity |  | |  |  |  |  |  |  |  |  |
| Black | 0.11 | | [-0.12, 0.32] | 83.80% | 0.12 | [-0.11, 0.34] | 84.85% | 0.14 | [-0.08, 0.36] | 88.35% |
| Hispanic, Latino or Spanish | 0.03 | | [-0.19, 0.24] | 59.55% | 0.02 | [-0.20, 0.25] | 57.95% | 0.05 | [-0.16, 0.26] | 66.30% |
| American Indian or Alaskan Native | 0.49 | | [-0.02, 1.02] | 96.78% | 0.47 | [-0.04, 0.99] | 96.47% | 0.47 | [-0.04, 0.98] | 96.40% |
| Asian race/ethnicity | 0.29 | | [-0.02, 0.59] | 96.90% | 0.29 | [-0.02, 0.60] | 96.80% | 0.29 | [-0.01, 0.60] | 96.95% |
| Pubertal timing | 0.13 | | [ 0.01, 0.26] | 98.32% | 0.3 | [-0.13, 0.72] | 90.67% | 0.27 | [-0.17, 0.70] | 88.15% |
| Boundary separation | -0.03 | | [-0.15, 0.09] | 68.73% | -0.02 | [-0.14, 0.09] | 65.58% | -0.02 | [-0.14, 0.10] | 63.85% |
| Pubertal timing *x* boundary separation |  | |  |  | -0.07 | [-0.25, 0.11] | 78.08% | -3.92E-03 | [-0.20, 0.19] | 51.95% |
| Male *x* pubertal timing *x* boundary separation | | |  |  |  |  |  | -0.08 | [-0.19, 0.02] | 93.85% |
| Emotional Stroop | | | | | | | | | | |
|  | | Main Effects | | | Moderation | | | 3-Way Moderation | | |
|  | | **Median** | **95% CI** | **PD** | **Median** | **95% CI** | **PD** | **Median** | **95% CI** | **PD** |
| (Intercept) | | -0.05 | [-0.80, 0.65] | 56.30% | -0.07 | [-0.82, 0.69] | 56.88% | -0.06 | [-0.77, 0.65] | 56.88% |
| Male | | -0.14 | [-0.30, 0.02] | 95.15% | -0.13 | [-0.31, 0.03] | 94.25% | -0.12 | [-0.29, 0.04] | 93.12% |
| Age | | 0.0012 | [-0.04, 0.04] | 52.48% | 1.27E-04 | [-0.04, 0.04] | 50.20% | -1.49E-03 | [-0.04, 0.04] | 53.23% |
| Income to needs | | 0.04 | [-0.04, 0.12] | 87.78% | 0.05 | [-0.03, 0.12] | 88.50% | 0.04 | [-0.03, 0.11] | 87.60% |
| College graduate | | -0.22 | [-0.41, -0.03] | 98.80% | -0.23 | [-0.43, -0.04] | 98.72% | -0.22 | [-0.41, -0.04] | 98.95% |
| Race/ethnicity | |  |  |  |  |  |  |  |  |  |
| Black | | 0.15 | [-0.07, 0.38] | 91.60% | 0.14 | [-0.08, 0.37] | 89.75% | 0.18 | [-0.04, 0.40] | 94.62% |
| Hispanic, Latino or Spanish | | 0.02 | [-0.19, 0.24] | 57.90% | 8.39E-03 | [-0.20, 0.23] | 52.88% | 0.04 | [-0.16, 0.26] | 66.40% |
| American Indian or Alaskan Native | | 0.51 | [ 0.01, 1.00] | 97.82% | 0.51 | [ 0.01, 1.02] | 97.65% | 0.52 | [ 0.01, 1.01] | 97.78% |
| Asian race/ethnicity | | 0.32 | [ 0.02, 0.63] | 98.52% | 0.32 | [ 0.02, 0.62] | 98.08% | 0.31 | [ 0.01, 0.61] | 97.92% |
| Pubertal timing | | 0.13 | [ 0.01, 0.25] | 98.17% | 0.29 | [-0.38, 0.97] | 79.65% | 0.37 | [-0.30, 1.03] | 85.80% |
| Boundary separation | | 0.21 | [-0.11, 0.55] | 90.40% | 0.23 | [-0.10, 0.58] | 90.85% | 0.21 | [-0.10, 0.54] | 90.55% |
| Pubertal timing *x* boundary separation | |  |  |  | -0.1 | [-0.51, 0.31] | 68.33% | -0.04 | [-0.45, 0.35] | 58.17% |
| Male *x* pubertal timing *x* boundary separation | | |  |  |  |  |  | -0.17 | [-0.32, -0.03] | 99.40% |

*Note:* Values are Bayesian posterior medians with 95% credible intervals. PD = probability of direction; ROPE = region of practical equivalence, defined as [−0.04, 0.04]; % in ROPE = percentage of the posterior distribution within the ROPE; ESS = effective sample size.
